# The Synthesis, Characterization, and Biological Evaluation of a Fluorenyl-Methoxycarbonyl-Containing Thioxo-Triazole-Bearing Dipeptide: Antioxidant, Antimicrobial, and BSA/DNA Binding Studies for Potential Therapeutic Applications in ROS Scavenging and Drug Transport

**DOI:** 10.3390/biom15070933

**Published:** 2025-06-26

**Authors:** Lala Stepanyan, Tatevik Sargsyan, Valentina Mittova, Zurab R. Tsetskhladze, Nino Motsonelidze, Ekaterine Gorgoshidze, Niccolò Nova, Monika Israyelyan, Hayarpi Simonyan, Franco Bisceglie, Lusine Sahakyan, Karapet Ghazaryan, Giovanni N. Roviello

**Affiliations:** 1 Scientific and Production Center “Armbiotechnology” NAS RA, 14 Gyurjyan Str., Yerevan 0056, Armeniatatev-sargsyan@ysu.am (T.S.); lusine_sahakyan@ysu.am (L.S.); 2 Institute of Pharmacy, Yerevan State University, 1 Alex Manoogian Str., Yerevan 0025, Armenia; 3 Faculty of Medicine, University Geomedi, 4 King Solomon II Str., 0114 Tbilisi, Georgia; valentina.mittova@geomedi.edu.ge (V.M.); zurab.tsetskhladze@geomedi.edu.ge (Z.R.T.); ekaterine.gorgoshidze@geomedi.edu.ge (E.G.); 4 Department of Chemical Science, Life and Environmental Sustainability, University of Parma, Parco Area delle Scienze 17/A, 43124 Parma, Italy; niccolo.nova@studenti.unipr.it (N.N.);; 5 Institute of Biostructures and Bioimaging (IBB), Italian National Council for Research (CNR), Area di Ricerca Site and Headquarters, Via Pietro Castellino 111, 80131 Naples, Italy

**Keywords:** fluorenyl-methoxycarbonyl, fmoc, thioxo-triazole, antioxidant properties, antimicrobial activity, bovine serum albumin, BSA, DNA modulation, bioactive peptides, Caucasus-endemic plants

## Abstract

We report on the synthesis and characterization of a novel fluorenyl-methoxycarbonyl (Fmoc)-containing thioxo-triazole-bearing dipeptide **5**, evaluated for potential therapeutic applications. The compound was tested for its antioxidant and antimicrobial properties, demonstrating significant effects in scavenging reactive oxygen species (ROS) and inhibiting microbial growth, particularly when combined with plant extracts from an endemic *Peonia* species from the Caucasus. Circular dichroism (CD) binding studies with bovine serum albumin (BSA) and calf thymus DNA revealed important interactions, suggesting the dipeptide’s potential in biomedically relevant conditions that involve DNA modulation. Molecular docking and CD spectra deconvolution provided additional insights into the binding mechanisms and structural characteristics of the formed complexes with the biomolecular targets. The Fmoc group enhances the dipeptide’s lipophilicity, which may facilitate its interaction with cellular membranes, supporting efficient drug delivery. A computational evaluation at the ωB97XD/aug-cc-pVDZ level of theory was carried out, confirming the experimental results and revealing a powerful potential of the peptide as an antioxidant, through FMOs, MEP analysis, and antioxidant mechanism assessments. Together, these findings suggest that this dipeptide could be valuable as an antimicrobial and antioxidant agent, with potential applications in pathologies involving oxidative stress, DNA modulation, and microbial infections.

## 1. Introduction

In recent years, the design and synthesis of bioactive peptides have emerged as a promising strategy in the development of novel therapeutic agents, particularly for combating oxidative stress, microbial infections, and diseases involving DNA damage. In fact, peptides play fundamental roles in numerous biological processes and have garnered significant interest in medicinal chemistry due to their broad spectrum of pharmacological activities, including antimicrobial, antioxidant, and enzyme-inhibitory properties. Their high specificity and biocompatibility make them promising candidates for therapeutic applications, particularly in infectious diseases, cancer, and neurodegenerative disorders [1,2,3,4,5,6,7,8,9,10]. As for their molecular components, aspartic acid and its derivatives play a crucial role in enzymatic catalysis, metal ion coordination, and the structural stabilization of peptides. Aspartic acid’s ability to form strong hydrogen bonds and coordinate with metal ions enhances the stability and interaction of peptides with biological targets, which is essential for their bioactivity [11,12,13,14].

### 1.1. Artificial Amino Acids and Their Role in Bioactive Peptides

Artificial amino acids, in addition to natural residues, provide a wide range of structural and functional properties that enhance the antioxidant and biomolecular binding ability of peptides by allowing the incorporation of specific molecular moieties. For example, triazoles, due to their strong biological activity, including enzyme inhibition, antimicrobial effects, and metal ion coordination, are increasingly being explored in drug design [15,16,17]. Incorporating triazole-containing compounds, especially thioxo triazoles, into peptide structures has emerged as a powerful strategy to enhance bioactivity, stability, and therapeutic potential. The thiotriazole moiety, in particular, exhibits enhanced pharmacological properties that can increase the efficacy of peptides as next-generation antimicrobial agents [18,19,20]. Incorporating triazole-containing amino acids into peptides can not only improve antimicrobial activity but also enhance the overall stability and interaction of the peptides with their targets [21,22]. This is particularly relevant in the development of antimicrobial peptides, where the combination of triazoles with anionic properties can further strengthen antibacterial activity. The presence of cations such as Zn^2^⁺ can synergistically interact with these peptides, improving their efficacy against both Gram-positive and Gram-negative bacteria [23,24]. In summary, peptides that combine aspartic acid with triazole-containing amino acids offer a promising approach for designing bioactive molecules. Natural sources of bioactive compounds, such as medicinal plants, have garnered increasing attention for their potential synergistic effects when combined with synthetic molecules [25,26,27].

### 1.2. Synergistic Effects of Synthetic Peptides and Bioactive Plant Compounds

This idea served as the basis for initiating a study on the synergistic properties of compounds synthesized by us and bioactive substances of plant origin. *Paeonia daurica* subsp. *mlokosewitschii* (Lomakin), an endemic plant of the Caucasus [28], was selected for this study due to its high biological potential and significant antioxidant activity [29]. Plants of the genus Paeonia are rich in bioactive compounds and widely used in traditional medicine. Thus, the high content of flavonoids, polyphenols, and monoterpene glycosides and anti-inflammatory, antiviral, and antibacterial therapeutic activities were demonstrated for *Paeonia lactiflora* Pallas and *Paeonia veitchii* Lynch root extracts [30]. Remarkably, antibacterial, antifungal, and anticoagulant properties and airway-relaxant, lipoxygenase-, and beta-glucuronidase-inhibiting activities were shown for *Paeonia emodi* root oil [31]. Moreover, extracts from fruit pods and the seeds of *Paeonia rockii* exhibited strong antibacterial effects against *Staphylococcus aureus* and *Proteus vulgaris* [32], while wound-healing properties and antibacterial activity were revealed for petal extracts of *Paeonia peregrina* Mill [33]. On the other hand, leaf extracts of representatives of tree peonies of *Paeonia* Section *Moutan* were characterized by a high terpenoid and flavonoid content and strong antibacterial capacities against food-borne and skin pathogens [34]. Seed kernel extracts of *Paeonia ostii* showed agonistic activity on cannabinoid receptors CB1 and CB2, indicating their potential use in the treatment of neurodegenerative diseases [35].

### 1.3. Aim of This Work

**Scheme 1:**
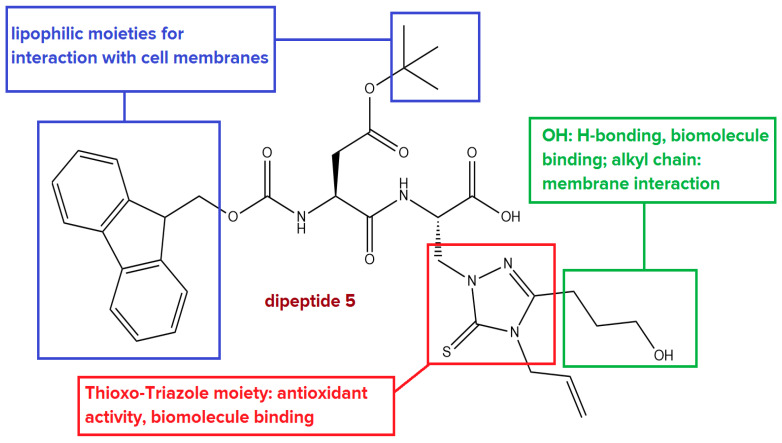
Schematic representation of the rationale for the structural moieties in our Fmoc dipeptide **5**.

In summary, the design, synthesis, and study of synthetic peptides have shown considerable promise in various therapeutic applications, particularly for enhancing biomolecular binding, antioxidant activity, and cell membrane interactions. In this study, we present the synthesis and characterization of a novel dipeptide, (*S*)-2-((*S*)-2-((((9H-fluoren-9-yl)methoxy)carbonyl)amino)-4-(tert-butoxy)-4-oxobutanamido)-3-(4-allyl-3-(3-hydroxypropyl)-5-thioxo-4,5-dihydro-1H-1,2,4-triazol-1-yl)propanoic acid, herein indicated as **5**, designed for potential therapeutic applications, incorporating key structural features to impart multifunctional properties. Specifically, the dipeptide was designed to include a thioxo-triazole moiety and an OH-containing alkyl chain to achieve antioxidant ROS scavenging and biomolecular binding capabilities, as well as Fmoc and tert-butyl groups for membrane binding and stability (Scheme 1). Specifically, the dipeptide was designed to possess the following key properties:

1. Antioxidant activity: The thioxo-triazole moiety could neutralize reactive oxygen species (ROS) contributing to the peptide’s antioxidant properties. This feature is particularly important in combating oxidative stress.
2. Biomolecular binding: The thioxo-triazole group enables binding to essential biomolecules, including proteins and nucleic acids, through metal ion coordination and other molecular interactions, potentially offering therapeutic benefits.
3. Cell membrane binding: The Fmoc and tert-butyl groups contribute to the peptide’s lipophilicity, enhancing its interaction with cell membranes. These groups help to facilitate the membrane penetration required for effective cellular uptake, improving bioavailability and overall activity.
4. Protection from degradation: The Fmoc and tert-butyl groups act as protective groups, not only ensuring controlled peptide synthesis but also shielding the dipeptide backbone from chemical and enzymatic degradation, thereby improving the stability and longevity of the compound in biological systems.

Thus, dipeptide **5** was designed to combine the synergistic effects of these structural features, with the aim of providing a highly stable, bioactive peptide with significant potential for antioxidant and biomolecular interaction applications. The inclusion of protective groups also ensures the peptide’s resilience against degradation, which is crucial for its effectiveness in long-term therapeutic use.

## 2. Materials and Methods

### 2.1. Materials

The materials used in this study included dimethylsulfoxide (DMSO), DCC, N-hydroxysuccinimide, NaHCO_3_, Na_2_CO_3_, NaOH, C_6_H_14_, C_4_H_8_O_2_, CH_3_COOC_2_H_5_, (*S*)-2-((((9H-fluoren-9-yl)methoxy)carbonyl)amino)-4-(tert-butoxy)-4-oxobutanoic acid (**1**), CH_2_Cl_2_, DPPH (2,2-diphenyl-1-picrylhydrazyl), ammonium molybdate, and methanol, which were purchased from Sigma Aldrich (St. Louis, MO, USA). The LB broth and LB agar plates for *E. coli* were purchased from LLC Elavia Biopreparations, Tbilisi, Georgia. The non-proteinogenic amino acid (*S*)-3-(4-allyl-3-(3-hydroxypropyl)-5-thioxo-4,5-dihydro-1H-1,2,4-triazol-1-yl)-2-aminopropanoic acid (**4**) was synthesized at the Armbiotechnology Scientific and Production Center (SPC) of the National Academy of Sciences of the Republic of Armenia (NAS RA). All chemicals were obtained from commercial suppliers and used without further purification. All solvents were freshly distilled before use.

### 2.2. Analytical and Spectroscopic Characterization Methods

Characterization data (Figures S1–S5) were obtained using the following analytical and spectroscopic methods. Thin-layer chromatography (TLC) was performed on Merck aluminum foil-backed sheets precoated with 0.2 mm Kieselgel 60 F254 (Darmstadt, Germany). The proton (^1^H) and carbon-13 (^13^C) nuclear magnetic resonance (NMR) spectra were recorded on a Varian Mercury 300 MHz spectrometer, using tetramethylsilane (TMS) as the internal standard (Palo Alto, CA, USA). Melting points were determined using an electrothermal apparatus (Bibby Scientific, Stone, UK). LCMS analysis was performed by Shimadzu LCMS 2020 with prominence-I LC-2030C 3D. IR characterization: The infrared analysis made use of an ATR (Attenuated Total Reflection) accessory, which allowed us to conduct a direct examination of the powder samples using an IRTracer-100 instrument (Shimadzu, Kyoto, Japan) using a KBr prism (4000–350 cm^−1^) with single reflection, at a resolution of 4 cm^−1^. Elemental analysis was performed using a Euro EA3000 elemental analyzer (Eurovector, Pavia, Italy).

### 2.3. Synthesis of the N-Oxysuccinimide Ester ***3***

Compound **1** (0.5g, 1.22 mmol) was dissolved together with N-hydroxysuccinimide (0.15 g, 1.32 mmol) in 2.0 mL of dioxane and 2.0 mL of methylene chloride, and to this solution, a solution of DCC (0.27 g, 1.31 mmol) in 2.0 mL of dioxane was added dropwise at 0 °C. The reaction was maintained at 0 °C for 1 h, followed by an additional hour at room temperature. TLC monitoring (chloroform/ethyl acetate/methanol, 2:4:1) was used to track the reaction progress. Upon completion, the dicyclohexylurea (DCU) precipitate was removed by filtration, and the filtrate, which contained the succinimide ester intermediate, was concentrated under vacuum, yielding an oily mass. A 0.46 g 75% yield of intermediate compound **3** was obtained and was immediately utilized in the subsequent peptide synthesis.

### 2.4. Synthesis of the Dipeptide: (S)-2-((S)-2-((((9H-fluoren-9-yl)methoxy)carbonyl)amino)-4-(tert-butoxy)-4-oxobutanamido)-3-(4-allyl-3-(3-hydroxypropyl)-5-thioxo-4,5-dihydro-1H-1,2,4-triazol-1-yl)propanoic acid (***5***)

First, 0.042 g (0.5 mmol) of NaHCO_3_ was added to a solution of 0.315 g (1.1 mmol) of (*S*)-3-(4-allyl-3-(3-hydroxypropyl)-5-thioxo-4,5-dihydro-1H-1,2,4-triazol-1-yl)-2-aminopropanoic acid (**4**) in 2 mL of 0.5 M NaOH, and then 0.45 g (0.88) mmol of N-hydroxysuccinimide ester of 9-fluorenylmethoxycarbonyl-protected α-amino acid (**3**) in 2 mL of dioxane was added. The reaction mixture was stirred for 3 h at room temperature and then transferred to a separatory funnel, and 6 mL of ethyl acetate, 3 mL of 10% citric acid solution, and 0.2 g of NaCl were added. After intense stirring, the organic layer was separated and dried with magnesium sulfate, and the solvent was distilled off under vacuum at 50 °C. The residual matter was crystallized from a mixture of ethyl acetate and petroleum ether in a ratio of 1/3. As a result, a 70% yield of compound **5** was obtained.

### 2.5. Characterization of ***5***

The dipeptide (**5**) is a white crystalline substance with a melting point of 132–135 °C. Elemental analysis: Found (%) C, 60.22; H, 6.27; N, 10.16; Calc for C_34_H_41_N_5_O_8_S (%) C, 60.07; H, 6.08; N, 10.30. ESI MS (m/z): 680.49 (found) 679,78 (expected for [C_34_H_41_N_5_O_8_S] + H^+^).

ATR-IR, ν, cm^−1^: 3649.32 (NH); 3564.45 (NH); 3506.59 (OH); 1720.50 (C=O, acid), 1681.93 (C=C); 1562.34 (C=O carbamate); 1519.91 (C=N carbamate); 1446.61 (C=C, aromatic); 1357.89 (C=S), 1249.87 (C-O ether); 1122.57 (C-O acid); 1130.29 (C–N heterocyclic); 1080.14 (C-N heterocyclic). ^1^H NMR (DMSO/CCl_4_ 1/3, δ, p.p.m, Hz): 1.40 s (9H, 3CH_3_), 1.43 br (1H, OH), 1.80–1.89 m (2H, CH2), 2.49 dd (1H, J = 16.0, 8.8, CH′), 2.60–2.69 m (3H, CH2, CH″), 3.48 t (2H, J = 5.9, CH_2_), 4.17–4.29 m (3H, CH, CH_2_), 4.38 dd (1H, J = 13.8, 8.1, CH′), 4.57 dd (1H, J = 13.8, 5.2, CH″), 4.60–4.68 m (2H, CH_2_), 4.72–4.78 m (2H, CH_2_), 5.10 ddt (1H, J = 16.9, 1.2, 1.1, CH′), 5.18 ddt (1H, J = 10.3, 1.2, 1.1, CH″), 5.86 ddt (1H, J = 16.9, 10.3, 5.6, CH), 7.25–7.38 m (5H, Ar, NH), 7.65–7.71 m (2H, Ar), 7.74–7.77 m (2H, Ar), 8.00 d (1H, J = 7.6, NH), 10.44 br s (1H, COOH). 13C: 21.1, 27.6 (3CH3), 28.5, 37.4, 43.0, 45.8, 46.6, 48.4, 51.2, 59.3, 71.9, 79.6, 117.3, 119.4, 125.1, 126.6, 127.1, 130.9, 140.5, 151.0, 155.3, 166.6, 168.9, 170.2, 171.1, 175.2.

### 2.6. Circular Dichroism

CD spectra were recorded using a Chirascan™ V100 spectropolarimeter (Applied Photophysics, Leatherhead, Surrey, UK) equipped with a Peltier Quantum accessory. The experiments were performed at 15 °C in phosphate-buffered saline (PBS) 10 mM with a pH of 7.4. The compounds **1**, **2**, **3**, and **4** mentioned in this manuscript are intermediates used exclusively for the synthesis of the final designed bioactive peptide, compound **5**. These intermediates were not tested as individual ligands. Compound **5** was prepared as a stock solution at a concentration of 9 mM in acetonitrile. For the measurements, 1.8 10^−2^ nmol of bovine serum albumin (BSA) (Merck, Darmstadt, Germany) and 9 nmol of compound **5** were used. For DNA binding, 1.54 nmol of DNA and 90 nmol of dipeptide were used. The final volume after mixing was 0.15 mL for BSA and 0.16 mL for DNA, with an optical path length of 0.5 mm, using an Applied Photophysics quartz cell (Applied Photophysics, Leatherhead, Surrey, UK). Each spectrum was obtained as an average of three scans. All CD spectra for DNA and BSA complexes with **5** were obtained by subtracting the CD spectrum of **5** alone, so the displayed complex CD spectra reflect the CD spectral properties due to the interactions between DNA, BSA, and compound **5**.

#### CD Deconvolution

To analyze the CD spectra, CD3 software (available online http://lucianoabriata.altervista.org/jsinscience/cd/cd3.html, accessed on 8 April 2025) [36] was used, where the CD (mdeg) and wavelength (nm) data were entered. For the analysis of protein structure, only data with positive coefficient values were selected, and the ‘Fit alpha beta coil’ option was applied.

### 2.7. Ligand Preparation and Molecular Docking

The dipeptide structure for docking was prepared through several steps. First, the SMILES string (C1C2=C(C3C(C2COC(N([H])[C@@](CC(OC(C)(C)C)=O)C(N([H])[C@@](CN2C(=S)N(CC=C)C(CCCO[H])=N2)C(O[H])=O)=O)=O)=CC=CC=3)C=CC=1) was retrieved from MolView by inputting the IUPAC name. This string was then pasted into Molinspiration (https://www.molinspiration.com/, accessed on 7 April 2024), where the 3D structure was generated using the Galaxy 3D Structure Generator v2023.08. The generated Molfile text was saved as a TXT file and converted into a PDB file using the Cactus Translator (https://cactus.nci.nih.gov/translate/, accessed on 7 April 2024). The resulting PDB file was edited in Discovery Studio Biovia, where hydrogen atoms were added, and energy minimization was performed using the CHARMM force field. The structure was uploaded to HDOCK for blind docking, selecting either 1BNA (ctDNA) or 4F5S (BSA) as the target PDB ID. We set standard parameters for molecular docking; more details on HDOCK software can be found at the following link: http://hdock.phys.hust.edu.cn/ (v1.1, Huazhong University of Science and Technology, China; accessed on 7 April 2024). Using the iterative, knowledge-based scoring function ITScore-PP, the HDOCK server evaluated and ranked the top ten docking poses obtained from each run. The energy score (HDOCK score) produced by ITScore-PP is dimensionless, where more negative values reflect stronger binding interactions between the ligand and the target macromolecule. These scores have been shown to correlate with experimental binding affinities, with a reported correlation coefficient of R = 0.71 [37]. Detailed information on the HDOCK docking server and its docking procedures can be found at http://hdock.phys.hust.edu.cn (accessed on 30 May 2022). We focused on analyzing the highest-ranked pose (Top-1), based on the energy scores provided by the software, as outlined in the Results and Discussion Section. The protein–ligand interaction diagrams featured in this study were generated using ProteinsPlus (https://proteins.plus/, accessed on 30 May 2022) [38].

### 2.8. Computational Analysis of Antioxidant Properties of ***5***

Over the past few decades, theoretical calculations using DFT have emerged as a powerful tool for clarifying the antioxidant properties of compounds [39,40,41,42,43]. All the computational assessments were performed on Gaussian 16 [44] and the GaussView 6 interface. All peptide geometry structures were optimized by the DFT method through the CAM-B3LYP/6-31 + g(d,p) level of theory; polarization and diffuse functions were chosen to denote a better accuracy toward polar bonds and because of the sulfur presence, respectively [45]. Moreover, geometries were optimized under methanol and DMSO solvation conditions using the polarizable continuum solvation model (CPCM) [46]. Subsequently, single-point energy and frequency calculations were performed on these geometries using ωB97XD/aug-cc-pVDZ adding the same solvation conditions. The distribution of charges into the molecule surface was clarified by a molecular electrostatic potential (MEP) map [47]. The MEP map plots electrostatic potential (ESP) at each point of the molecular surface, which is influenced by all the nuclei and electrons of this molecule and depends on the distance of them from the evaluated point; in this way, nuclei contribute positively and electrons negatively, as the equation in the Supplementary Materials reports for a generic vector position.

FMOs were assessed by evaluating HOMO/LUMO gaps in each condition to interpret the experimental results, and additionally, the following equations were used to determine these descriptors, using the IP (ionization potential) and EA (electron affinity) [47,48]:

$$Chemical\;hardness\;\eta =\frac{\left(IP-EA\right)}{2}=\frac{-E_{HOMO}+E_{LUMO}}{2}$$

$$Chemical\;potential\;\mu =-\frac{IP+EA}{2}=\frac{E_{HOMO}+E_{LUMO}}{2}$$

$$Chemical\;softness\;\sigma =\frac{1}{\eta }$$

$$Electronegativity\;\chi =-\frac{E_{HOMO}+E_{LUMO}}{2}=-\mu$$

$$Electrophilicity\;\omega =\frac{\mu^{2}}{2\eta }$$

TD-SCF calculation at the ωB97XD/aug-cc-pVDZ level was run in MeOH solvation to compare transitions with the UV-vis spectrum [49].

With regard for the two possible radical scavenging mechanisms of DPPH toward the peptide, single-electron transfer (SET) and hydrogen atom transfer (HAT), an assessment along these channels was carried out in the gas phase and under solvation [40,41,42,50,51]. For the HAT mechanism, BDE (bond dissociation energy) was calculated toward the alcoholic group, optimizing the geometry of the R-O· radical in the doublet state, while the SET mechanism was evaluated in terms of IP (ionization potential energy):

ArOH+DPPH·→ArO·+DPPH-H

BDE_OH_ = H(ArO·) + H(H·) − H(ArOH)

$$\mathrm{ArOH}\to {\mathrm{ArOH}\cdot }^{+}+e^{-}$$

IP = H(ArOH·^+^) − H(ArOH)

### 2.9. Antioxidant and Antibacterial Activity of Peptide ***5***

#### 2.9.1. Plant Material

*Paeonia daurica* subsp. *mlokosewitschii* (Lomakin) D. Y. Hong (family Paeoniaceae) plants were collected in July 2024 in the National Botanical Garden of Georgia (41°41′11.9″ N 44°48′10.4″ E, Tbilisi, Georgia).

#### 2.9.2. Drying Processes

Leaves and roots (5 g) were freeze-dried using a DW-10N freeze dryer (Drawell, Chongqing, China) in a 500 mL vacuum flask at 10 Pa and a final condenser temperature of −55 °C, until the plant material reached a constant weight, as determined by measuring the dry weight.

#### 2.9.3. Extraction of Plant Samples

The extraction efficiencies of different solvents for leaf and root plant material were tested earlier, and methanol was demonstrated to be the most efficient solvent [52,53]. The roots were homogenized in a ratio of 1:5 with 80% methanol, followed by continuous stirring for 24 h at room temperature using an orbital shaker at 270 rpm. The crude extracts were clarified by centrifugation at 5000× *g* for 15 min (TD6 Benchtop Centrifuge, Drawell, China). Leaf and root extracts obtained using 80% methanol were rotary-evaporated at 50 °C using a DW-ORE2000 Rotary evaporator (Drawell, Chongqing, China), and residues were dissolved in 80% DMSO.

#### 2.9.4. DPPH Radical Scavenging Activity Assay

The stock solutions of dipeptide **5** (10 mg/mL) were prepared in either 99.9% DMSO or 99.9% methanol.

The DPPH radical scavenging activity of dipeptide **5** was measured according to [54]. Dipeptide **5** solutions (200 μL at different concentrations) were mixed with 2.8 mL of 0.1 M DPPH solution in 99.9% methanol to a final concentration of 50–500 μg/mL. The range of dipeptide **5** concentrations for this assay was chosen based on preliminary experiments. After incubation at room temperature for 30 min in the dark, the absorbance of the resulting solution was measured at 517 nm using a DU-8800 RS spectrophotometer (Drawell, Chongqing, China). The blank was a methanolic dilution of DPPH, and the absorbance of the blank was 0.98 ± 0.02. The scavenging activity was estimated based on the percentage of DPPH radical scavenged according to the following equation:

Scavenging effect (%) = [(control absorbance − sample absorbance)/(control absorbance)] × 100

The concentrations of dipeptide **5** required for 50% inhibition (IC_50_) were calculated by plotting the concentrations of dipeptide **5** versus the inhibition of DPPH (%), and the data were fit with a straight line (linear regression).

Y = a × X + b,

IC_50_ = (50 − b)/a

#### 2.9.5. Total Antioxidant Capacity (Phosphomolybdate Assay)

The total antioxidant capacity (TAC) of dipeptide **5** was determined by the phosphomolybdate method using gallic acid as a standard [55]. Dipeptide **5** solutions (200 μL at different concentrations) were mixed with 2.8 mL of reagent solution (0.6 M sulfuric acid, 28 mM sodium phosphate, and 4 mM ammonium molybdate) to a final concentration of 50–650 μM. The range of dipeptide **5** concentrations for this assay was chosen based on preliminary experiments. The tubes were capped and incubated in a water bath at 95 °C for 90 min. After the samples had cooled to room temperature, the absorbance of the mixture was measured at 765 nm against a blank, and the results are expressed in terms of gallic acid equivalent (GAE). A typical blank contained 2.8 mL of the reagent solution and 200 μL of the solvent and was incubated under the same conditions.

#### 2.9.6. Determination of Antibacterial Activity

The Kir–Bauer diffusion method was employed for antibacterial activity screening [56]. The *Escherichia coli* ATCC 25,922 strain was used in this study. The bacteria were grown in an LB broth for 16–18 h at 37 °C (10^9^–10^10^ CFU/mL). Sterile blank disks with a diameter of 6 mm were individually placed on an LB nutrient agar plate covered with 300 μL of the bacterial strain. Ten microliters of the dipeptide **5** solution (5–50 μg/disk) in DMSO were applied on the disk. In experiments with plant extract, *Paeonia daurica* subsp. *mlokosewitschii* leaf or root extracts (200 μg/disk) were applied on the disk. To assess the effect of dipeptide **5** on the antibacterial activity of *Paeonia daurica* subsp. *mlokosewitschii* extracts, different concentrations of dipeptide **5** (12–25 μg/disk) were mixed with either leaf or root extracts (200 mg/disk) and applied on the disk, and the volume of the solution per disk in all variants was 10 mL. These plates were incubated at 37 °C for 24 h. The antimicrobial activity was determined in triplicate by measuring the diameter of the inhibition zone (mm). Amoxicillin (30 μg/disk) was used as the positive control. Dimethyl sulfoxide (80%) was used as the negative control.

#### 2.9.7. Statistical Analysis

The analyses were conducted in triplicate, and the data obtained are expressed as the mean ± standard deviation. The statistical analysis of differences was performed using a *t*-test of variance with Microsoft Office Excel 365 software, and *p* < 0.05 was considered to indicate a statistically significant difference.

## 3. Results and Discussion

### 3.1. Synthesis of the Dipeptide

**Scheme 2:**
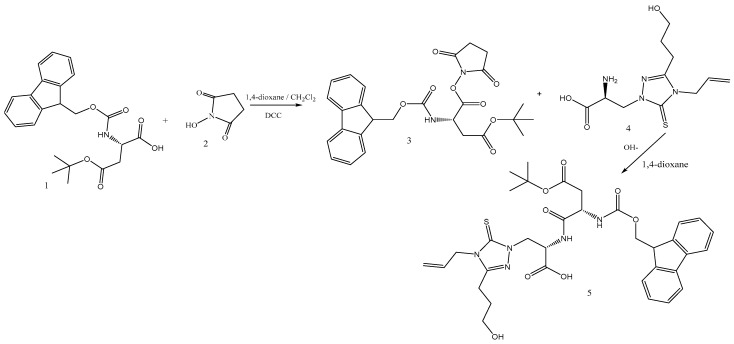
Schematic representation of the synthetic route to dipeptide **5**.

The synthesis of the dipeptide containing the non-protein amino acid (S)-3-(4-allyl-3-(3-hydroxypropyl)-5-thioxo-4,5-dihydro-1H-1,2,4-triazol-1-yl)-2-aminopropanoic acid (**4**) was performed following the procedure outlined in Scheme 2. Initially, the protected amino acid **1** underwent transformation into a succinimide ester. This was achieved by activating the carboxyl group of Fmoc-amino acid using N-hydroxysuccinimide (HOSu) (**2**), in the presence of dicyclohexylcarbodiimide (DCC) as the coupling reagent. The reaction was conducted in a solvent mixture of dioxane and methylene chloride, resulting in the stable formation of the N-hydroxysuccinimide ester **3**. In the subsequent step, condensation was performed by reacting **4** with the N-hydroxysuccinimide ester of Fmoc-amino acid (**3**). Different reaction media and molar ratios of sodium hydroxide (NaOH) to sodium carbonate (Na_2_CO_3_) were tested, with the optimum results obtained at a NaOH/Na_2_CO_3_ molar ratio of 1:0.5. Reactions conducted with NaOH alone, without Na_2_CO_3_, led to an increased formation of by-products. Consequently, the desired dipeptide **5** was successfully synthesized (Scheme 2).

The synthetic route to the dipeptide from Fmoc-amino acid **1** involved the activation of the carboxyl groups using HOSu and DCC. This method is highly regarded because of the stability of the intermediates, which allows for efficient coupling reactions with various amino acids. Through adjustments to the molar ratios of NaOH and Na_2_CO_3_, the outcome of the reaction can be fine-tuned. A critical finding from this experiment was the identification of an optimal molar ratio of 1:0.5 (NaOH: Na_2_CO_3_), which significantly minimized the formation of unwanted by-products. This approach not only ensured the successful synthesis of dipeptide **5** but also demonstrated the robustness and scalability of the method for larger-peptide-synthesis applications.

### 3.2. Preliminary Biological Studies

#### 3.2.1. Antioxidant Activity

**Figure 1:**
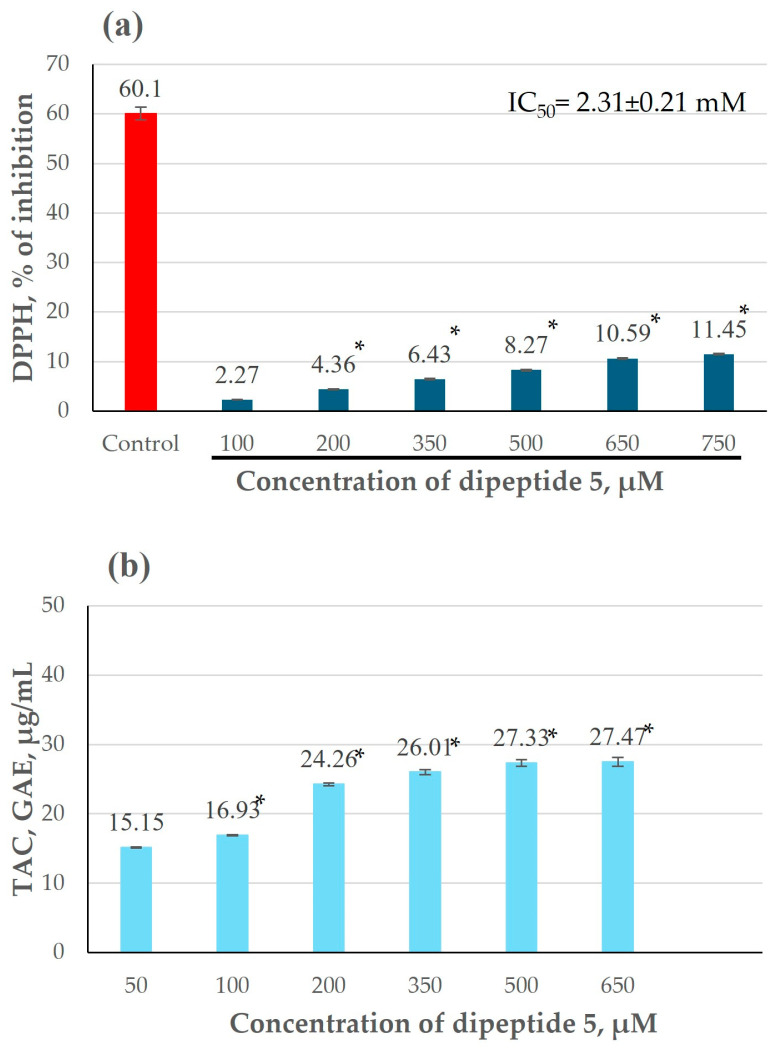
Antioxidant activity of dipeptide **5** solution in DMSO, evaluated using DPPH (**a**) and TAC methods (**b**). Gallic acid solution (1 µM) in DMSO was used as a control (red column). The values in each column represent the mean ± standard deviation of the mean of three independent experiments. Asterisks represent significant differences (*t*-test, * *p* < 0.05) when compared to the antioxidant activity with the lowest concentration of dipeptide **5** in the corresponding experiment.

Recognizing the critical role of peptides and amino acid-containing structures in the development of antioxidant compounds and antibacterial agents, we conducted preliminary investigations to evaluate the potential of dipeptide **5** in this regard. Its antioxidant activity was assessed using both the DPPH radical scavenging assay and the phosphomolybdate method, providing complementary insights into its capacity to neutralize free radicals and reduce oxidative stress (Figure 1).

Since dipeptide **5** is water-insoluble, two organic solvents, DMSO and methanol, were selected for determining antioxidant activity. These solvents were chosen to assess the impact of different solvents on the antioxidant activity of our compound [57]. DMSO was chosen because it has been shown to have no significant effect on the DPPH assay [58], while methanol was included as several studies have reported high antioxidant activity readings with this solvent when using the DPPH method [59,60].

Interestingly, the highest antioxidant activity was revealed for a 750 μM dipeptide **5** solution in DMSO (11.45%, Figure 1a), whereas the antioxidant activity of the same concentration of dipeptide in methanol was nearly half as low (5.99%, Figure S6). The IC_50_ value of the dipeptide **5** solution in DMSO was significantly lower than that of the dipeptide **5** solution in methanol (2.31 ± 0.21 and 3.41 ± 0.28 mM, respectively, Figure 1a and Figure S6a). A significant total antioxidant capacity (TAC) was observed for the dipeptide **5** solution in DMSO. For the 650 µM dipeptide **5** solution in DMSO, the highest TAC value detected was 27.47 ± 2.15 μg/mL, expressed as gallic acid equivalent (GAE, Figure 1b). The comparison of TAC values for dipeptide **5** in the two solvents revealed that the dipeptide **5** solution in methanol exhibited a significantly higher TAC than the DMSO solution (Figure 1b and Figure S6b). Specifically, the TAC for the 650 µM dipeptide **5** solution in methanol was 46.89 ± 3.66 GAE, μg/mL. The observed solvent-dependent antioxidant activity of dipeptide **5**, as measured by the DPPH and phosphomolybdate assays, likely reflects differences in solvent–peptide interactions and assay-specific redox mechanisms. The higher DPPH activity in DMSO suggests better radical scavenging efficiency in aprotic environments, possibly due to conformation or side-chain accessibility. Conversely, the higher TAC values observed for the methanol solution in the phosphomolybdate assay may reflect improved solvent compatibility, which enhances the peptide’s overall reducing capacity.

#### 3.2.2. Preliminary Antimicrobial Studies

Additionally, the antibacterial effects of dipeptide **5** were tested on *Escherichia coli* (*E. coli*), a widely used model for Gram-negative bacteria and one of the most common causes of bacterial infections, with growing concerns over antibiotic resistance [61,62]. These studies aimed to elucidate the dual functionality of dipeptide **5**, contributing to its broader applicability in therapeutic and biomedical contexts. The in vitro antibacterial activity of dipeptide **5** was tested based on the presence or absence of a zone of inhibition. The inhibition of *E. coli* growth by dipeptide **5** at different concentrations (5–50 μg/disk) was not observed (Figure S7a). However, dipeptide **5** had a different effect on the antibacterial activity of *Paeonia daurica* leaf and root extracts against *E. coli* (Figure S7b and Table S1). Thus, the addition of dipeptide **5** to *Paeonia daurica* leaf extracts increased the antibacterial activity of the extract. Bacterial growth inhibition increased as the concentration of dipeptide **5** increased in the mixture with the *Paeonia daurica* leaf extract, with the inhibition zone (IZ) increasing from 13.6 ± 0.6 (200 μg/disk *Paeonia daurica* leaf extract) to 15.5 ± 0.4 (200 μg/disk *Paeonia daurica* leaf extract + 25 μg/disk dipeptide **5**, Figure S7b, disks 1–3, and Table S1). On the contrary, the addition of dipeptide **5** to *Paeonia daurica* root extracts decreased the antibacterial activity of the extract. This can be seen by comparing the IZ values (IZ was 13.9 ± 0.9 for the 200 μg/disk *Paeonia daurica* root extract and 12.0 ± 1.2 for the 200 μg/disk *Paeonia daurica* root extract + 25 μg/disk dipeptide **5**, Figure S7b, disks 4–6, and Table S1). The results of the chemical characterization of the root extracts of three Peonia species (*Paeonia tenuifolia* L., *Paeonia peregrina* Mill., and *Paeonia officinalis* L.) showed that *Paeonia* terpenes, such as paeoniflorin derivatives, form the dominant class of molecules present in such extracts [63,64]. On the other hand, in *Paeonia officinalis* L. leaf extracts [65], gallic acid and its derivatives were the main components. The opposite effects of dipeptide **5** on the antibacterial activity of *Paeonia daurica* leaf and root extracts may be attributed to differential interactions with the predominant bioactive constituents in each extract, namely, gallic acid derivatives in the leaves and paeoniflorin-type terpenes in the roots. In the case of the leaf extract, dipeptide **5** could potentially form non-covalent interactions with gallic acid, such as hydrogen bonding or π-π stacking, which might enhance the solubility, stability, or membrane permeability of gallic acid [66], thereby contributing to increased antibacterial activity against *E. coli*. Conversely, in the root extract, dipeptide **5** may interfere with the activity of paeoniflorin-type compounds, perhaps by forming less-active complexes or by altering the overall extract environment in a manner that diminishes its effectiveness. These observations suggest that compound-specific interactions within the extract matrix may influence the outcome of antibacterial assays, though further studies are needed to clarify the underlying mechanisms. Additionally, further experiments are planned, including the investigation of the observed effect of dipeptide **5** on the antibacterial activity of *Paeonia daurica* extracts. These will involve determining the minimum inhibitory concentration (MIC) and biofilm inhibitory concentration (BIC) for *E. coli*, as well as exploring the effect on Gram-positive bacteria. 

### 3.3. CD DNA and BSA Binding Studies

As for the evaluation of the biomolecular interaction ability of our dipeptide, we employed circular dichroism (CD) spectroscopy to explore the interaction of dipeptide **5** with key biological models. Specifically, calf thymus DNA [67], a well-established model for double-stranded DNA (dsDNA), was utilized to assess the potential of dipeptide **5** to interact with and alter the structure of DNA. Additionally, we investigated its effects on serum albumins, which play a critical role in the transport of biomolecules through the bloodstream and the controlled release of drugs at target sites [68]. Bovine serum albumin (BSA) was selected as a representative model to evaluate these interactions due to its relevance in biomedical research [69].

**Figure 2:**
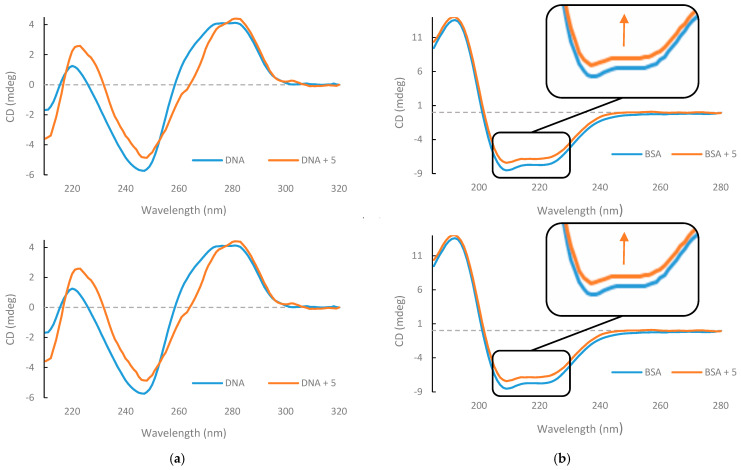
CD binding studies with the dipeptide as ligand and (**a**) DNA (ctDNA, 9.6 µM, phosphate buffer: pH of 7.4, 15 C) or (**b**) BSA (0.12 µM, phosphate buffer: pH of 7.4, 15 C). The arrows in the CD plot indicate the direction of variation; specifically, they point upward as the CD intensity decreases.

CD analysis revealed that dipeptide **5** induces notable structural changes in calf thymus DNA (Figure 2a). These changes are characterized by an increase in intensity around 225 nm, accompanied by broader modifications in the overall CD profile, particularly at approximately 225 nm and 270 nm. This observation highlights the ability of dipeptide **5** to interact with and modify the structure of DNA, which is crucial in regulating DNA-related processes, such as transcription and replication. Furthermore, structural alterations were observed in BSA, a key model for serum albumins, with pronounced changes in CD intensity at the minima located at 209 nm (ΔCD = 1.11188; %CD change = 13.04%) and 222 nm (ΔCD = 0.97343; %CD change = 12.63%) (Table S2).

Secondary structure deconvolution (Table 1) demonstrated that dipeptide **5** promotes an increase in the alpha-helix content of BSA by +10.16%, reflecting the induction of increased structural organization upon interaction with this peptide. This finding underscores the ability of the serum albumins to bind and transport dipeptide **5**, which holds significant potential for biomedical applications. Fortunately, the interaction exhibits balanced strength, as indicated by % CD changes ranging between 12.63% and 13.04% (Table S2). This moderate binding strength suggests that serum albumins could effectively release dipeptide **5** at target sites, facilitating its action as a potential therapeutic agent.

Overall, these findings provide encouraging evidence from spectroscopic analysis, suggesting that **5** could serve as a promising candidate for therapeutics targeting DNA function, as well as emphasizing the role of BSA as a transporter of the dipeptide, with implications for drug delivery.

### 3.4. In Silico Studies

**Figure 3:**
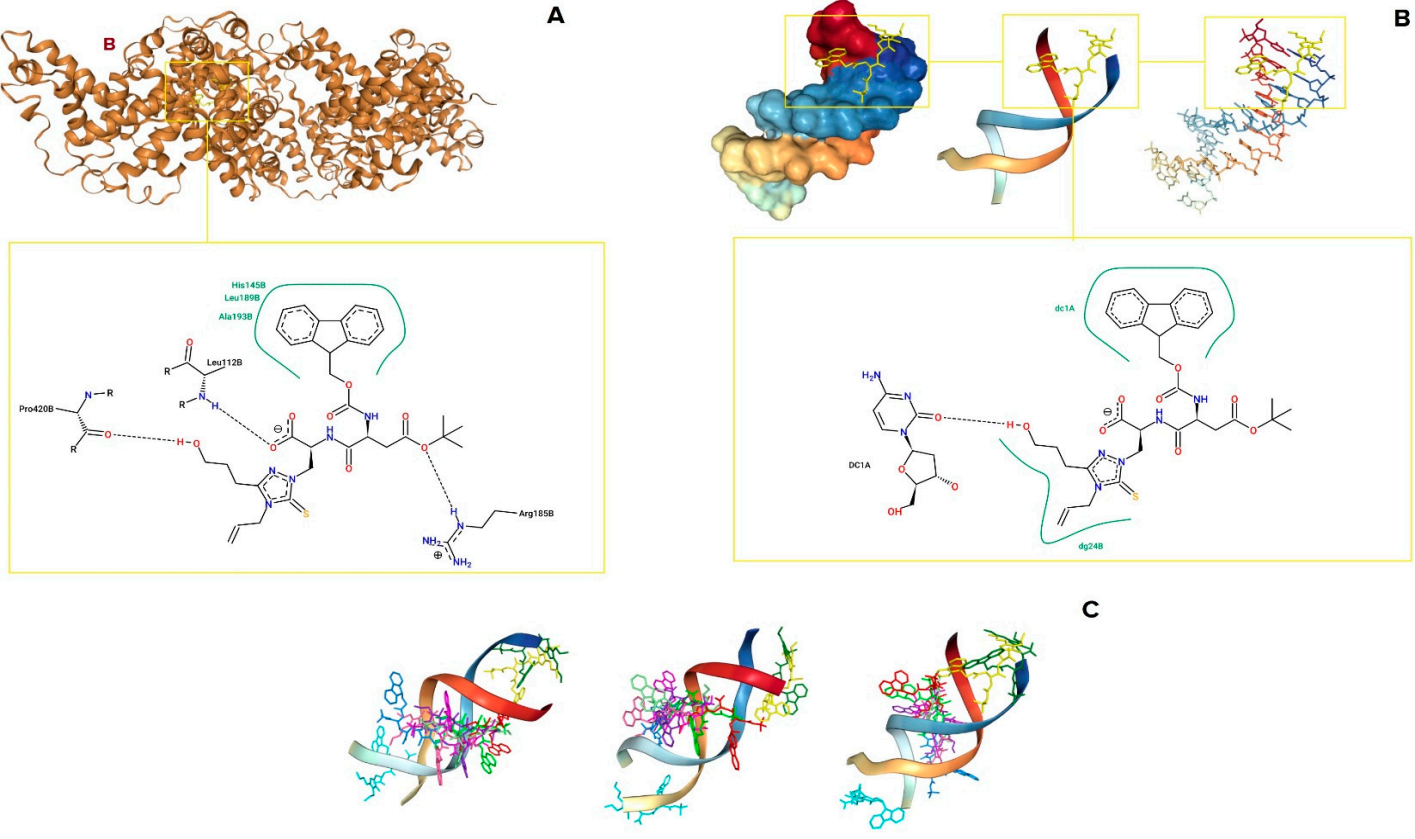
Molecular docking studies were performed with (**A**) BSA (PDB ID: 4F56) and (**B**) DNA (ctDNA, PDB ID: 1BNA). The ligand (dipeptide) is highlighted in yellow for clarity. The 2D interaction diagrams in Figure 4a,b were generated using ProteinPlus software (https://proteins.plus/, accessed on 7 April 2025). A multicolor representation of the ligand corresponding to the top ten poses from the docking is shown in panel (**C**), clearly illustrating how the dipeptide binds to the double-stranded DNA in the major groove.

Molecular docking simulations using HDOCK software (v1.1, Huazhong University of Science and Technology, China) [70] revealed that compound **5** binds effectively to BSA (PDB ID 4F5S) [71], residing within a cavity in chain B of the BSA dimer (Figure 3A). This interaction is characterized by both hydrophobic interactions and hydrogen bonding. Specifically, hydrogen bonds are formed with Leu112, Arg185, and Pro420, while hydrophobic interactions involve His145, Leu189, and Ala193 (Figure 3A). For DNA binding, we used a double-stranded DNA model of calf thymus ctDNA, corresponding to the PDB ID 1BNA [72]. The docking results predicted that compound **5** binds to the dC1 residue of chain A of dsDNA, engaging in both hydrogen bonding and hydrophobic interactions, as well as to dG24 of chain B. Remarkably, the hydroxy group in the alkyl-hydroxy moiety of the dipeptide acts as a hydrogen donor, contributing to the overall interaction with both BSA and dsDNA (Figure 3A,B), aligning with our expectations from the molecular design (Scheme 1). Similarly, the thioxo-triazole moiety plays a crucial role in the biomolecule binding, as observed with DNA (Figure 3B), confirming our rational design strategy (Scheme 1). Interestingly, even across all ten of the topmost favorable poses predicted by HDOCK, the ligand predominantly binds in the major groove of dsDNA, indicating its preference as a major groove binder (Figure 3C). From an energetic perspective, both serum albumin and dsDNA binding occur favourably, with HDOCK scores of −202.13 (average of top 1–10 poses: −183.52 ± 10.68) and −171.22 (average of top 1–10 poses: −151.40 ± 7.63), respectively, indicating stable complex formation (Table 2).

### 3.5. Computational Evaluation: MEPs, FMO Analysis and Global Reactivity Indexes, and Antioxidant Mechanism

**Figure 4:**
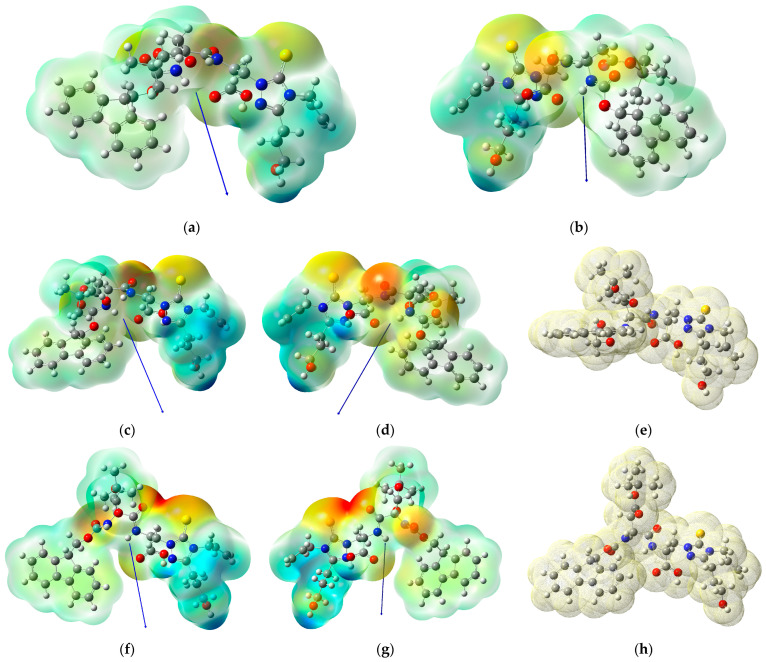
MEPs and dipole-moment vectors (represented by blue arrows) of the molecule on the front (**a**) and back (**b**) sides in the gas phase. Front side (**c**), back side (**d**), and solvation sphere (**e**) in MeOH. Front side (**f**), back side (**g**), and solvation sphere (**h**) in DMSO. MEP charge scales were normalized in the same range.

Different equilibrium geometries (see output coordinates in Supplementary Materials) were found for **5** in the gas phase and MeOH and DMSO solvation conditions. The gas-phase molecular electrostatic potential (MEP, Figure 4a,b) shows quite a homogeneous charge distribution on the molecule surface but also presents some polarized functional groups. This agrees with the capability of assuming different geometries and, subsequently, different solvation cavities between MeOH and DMSO, as reported in Figure 4c–h. From a biological standpoint, polarized groups can interact with cell membranes, whereas the incorporation of fluorene and aliphatic groups may enhance membrane permeability. This effect is attributed to changes in peptide geometry and, consequently, in its electrostatic surface potential as a function of the solvation environment. Although the molecule possesses high dipole-moment values (see Table 3), which suggests that it could need an active transporter to enter the cells, a Log(P) value of 3.31 was estimated, meaning possibly good permeability toward the cell membrane.

**Figure 5:**
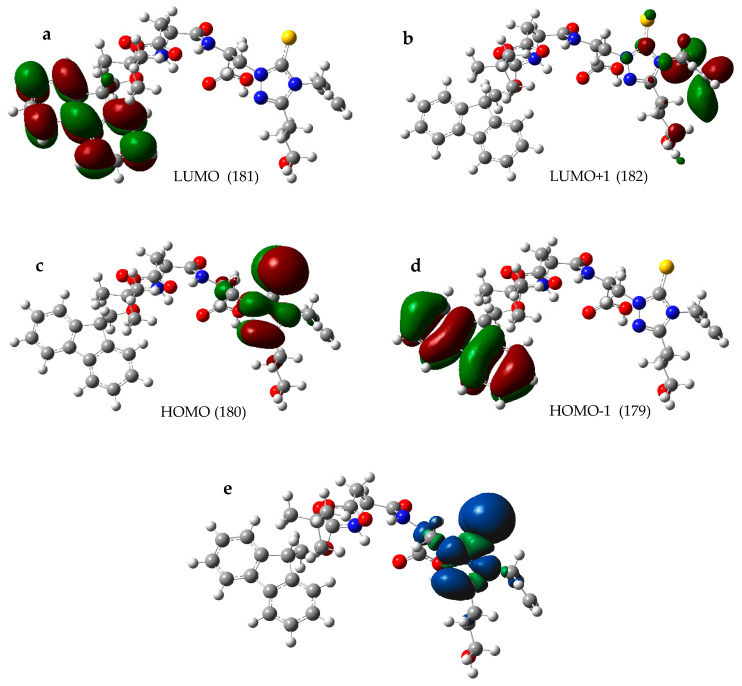
FMO depictions (**a**–**d**) and triplet-state spin density (**e**). These present the HOMO localized on the thioxo-triazole ring, which is involved in oxidation, while reduction takes place on the fluorene group (LUMO). HOMO-1 is involved in the $\pi \to \pi^{*}$ fluorene transition at 260 nm.

Frontier Molecular Orbitals (FMOs) help in having a better understanding of molecule reactivity and interaction with ROS [43,47]. In this way, the HOMO identifies the region or functional group of the peptide that tends to lose an electron when interacting with the scavenger, while the LUMO indicates the area where the reduction of the molecule is likely to occur. The FMOs of the peptide (Figure 5) were calculated in the gas phase; MeOH and DMSO solvation and energy gaps are reported in Table 3. As shown in Figure 5c, HOMO is localized onto the thioxo-triazole ring, where the maximum spin density (triplet state) is also present (Figure 5e), demonstrating that the scavenging of an electron by ROS, and by DPPH, in our case, takes place primarily on this ring. Conversely, the LUMO orbital is localized on the fluorene group, likely because its extensive conjugation can effectively stabilize a negative charge upon reduction. Reported FMOs have the same shape both in the gas phase and in the two solvents, changing only the gaps between each other; based on these results, a smaller HOMO/LUMO gap is observed in DMSO than in MeOH. This fact could suggest that the peptide is more reactive toward oxidation in DMSO and the expected RSAs% are bigger, which is consistent with the experimental results (Figure 1 and Figure S6).

Additionally, important reactivity descriptors were calculated including chemical hardness (η), chemical potential (μ), softness (σ), electronegativity (χ), and the electrophilicity index (ω), listed in Table 3. In terms of local indexes, a molecule is more stable when it is harder, while it is more reactive when it is softer. Parr et al. [43] suggested the electrophilicity of a molecule to be its affinity to soak up electrons; this global index quantifies the energy stabilization which occurs when a system gains an additional electronic charge from its environment.

By definition, it reflects both the system’s capacity to accept an extra electron as an electrophile and its resistance to exchanging electrons through the environment; on the other hand, electronegativity simply measures the ability to polarize an electronic cloud. Since electrophilicity integrates information about electron transfer (chemical potential) and stability (hardness), it is considered a good descriptor of overall chemical reactivity [47]. Based on the calculated indexes, the molecule appears softer and exhibits significantly higher electrophilicity, nearly double in DMSO, indicating an overall greater reactivity in this solvent, as also confirmed experimentally, despite the comparable electronegativity values observed in both solvents. The origin of this behavior can be attributed to differences in solvation conditions. Although dipeptide **5** displays similar electronegativity values, suggesting a comparable tendency to attract electrons, its optimized geometry in MeOH results in a higher dipole moment, thereby promoting stronger interactions with the solvent. Subsequently, MeOH stabilizes the peptide reactivity more than DMSO, in which the HOMO/LUMO gap decreases while the softness and electrophilicity index increase together with global reactivity. Time-Dependent Self-Consistent Field (TD-SCF) calculation predicted the UV-vis spectrum (Figure S8) with a singlet-state absorption at a 260 nm wavelength (4.77 eV, f = 0.40), which is associated with a $\pi \to \pi^{*}$ localized electron transition into the fluorene group, taking place between HOMO-1 (179) and LUMO (181) with 60% weight, and then an absorption at 240 nm (5.15 eV, f = 0.35), which is generated by the electronic transition from the HOMO to higher MOs with different weights. Comparing this with the experimental UV-vis spectrum in MeOH, we can consider the 260 nm absorption peak to be like the fluorene-localized electron transition and then the 234 nm absorption to be like the sulfur lone pair transition. Finally, we deemed it of interest to conduct a deeper investigation into the antioxidant interaction mechanism with DPPH. Given that the HOMO is primarily localized on the thioxo-triazole ring, we hypothesized that this moiety serves as the site for the SET (single-electron transfer) process. Furthermore, the presence of an alcoholic group, which is susceptible to oxidative hydrogen transfer, may provide an additional pathway for antioxidant activity. Thus, the oxidation mechanism assessment along these channels was performed in terms of bond dissociation energy (BDE)_OH_ for hydrogen atom transfer (HAT) and ionization potentials (IPs) for SET. As reported in Table 4, BDEs follow the same trend of HOMO/LUMO energy gaps and the hydrogen atom transfer prevails in all examined conditions, with significantly different energies reported with respect to electron transfers. Hence, DPPH oxidizes the dipeptide likely by the HAT mechanism. Furthermore, a more favorable pathway in DMSO is calculated with respect to MeOH, as experimentally observed and theoretically expected by FMO analysis.

## 4. Conclusions

Dipeptide **5** was evaluated in preliminary assays to assess its antioxidant and antimicrobial activities, both individually and in combination with plant extracts derived from a Caucasus-endemic species of *Paeonia*. Specifically, **5** enhanced the antibacterial activity of *Paeonia daurica* leaf extracts against *E. coli*, increasing the inhibition zone, while it reduced the activity of root extracts, potentially due to interactions with paeoniflorin-type terpenes in the roots. To further understand its biological interactions, we conducted circular dichroism studies and investigated its binding affinity to bovine serum albumin and calf thymus DNA, demonstrating significant DNA binding, involving the major groove of DNA as predicted by molecular docking, and an increase in the α-helical content in BSA. Moreover, molecular docking and CD spectral deconvolution were utilized to provide insights into binding mechanisms and structural characteristics. Computational evaluation through the DFT method confirmed the experimental results, reporting a good overall reactivity toward the DPPH radical in DMSO with respect to MeOH, as reported by energy gaps and reactivity indexes. FMO analysis suggested the thioxo-triazole ring to be the functional group where oxidation mainly takes place. Moreover, the MEPs provided insight into the potential peptide’s permeability across cell membranes. Additionally, scavenging by DPPH favors the HAT mechanism, and the TD-SCF-predicted UV-Vis spectrum is in good agreement with the experimental results. Collectively, our findings suggest that this novel dipeptide exhibits promising antimicrobial and antioxidant properties, with potential applications in oxidative stress-related diseases, DNA modulation, and antimicrobial resistance management in combination with plant extracts.

## Figures and Tables

**Table 1:** CD deconvolution of the spectra obtained from the BSA binding study.

Secondary Structure ^1^ Component	Rate Before Ligand Addition (%)	ΔSecondary Structure (%)
alpha-helix	65.49	+10.16
beta-sheet	13.10	−0.41
turn	4.66	+0.12
random coil	16.76	−9.87

^1^ Secondary structure analysis was conducted following CD deconvolution. ΔSecondary structure contribution values represent the relative changes in secondary structure components (alpha-helix, beta-sheet, turn, and random coil) upon interaction with dipeptide **5**. These values were calculated as the difference between the secondary structure contents of the BSA + dipeptide **5** complex and BSA alone.

**Table 2:** Summary of HDOCK docking results. For each target (PDB ID), the top-1 pose score, average ± SD of the top 10 poses, and key binding site residues are reported.

Target	PDB ID	HDOCK Score (Top-1 Ranked Pose)	HDOCK Score (Top 1–10 Poses, Average ± SD)	Residues Involved in Binding
BSA	4F5S	−202.13	−183.52 ± 10.68	Leu112, Arg185, Pro420 (hydrogen bonds); His145, Leu189, Ala193 (hydrophobic interactions)
dsDNA	1BNA	−171.22	−151.40 ± 7.63	dC1 (chain A), dG24 (chain B) (hydrogen bonds and hydrophobic interactions)

**Table 3:** HOMO/LUMO gaps, chemical hardness η, chemical potential μ, chemical softness σ, electronegativity χ, and global electrophilicity ω, calculated in gas phase, MeOH, and DMSO at ωB97XD/aug-cc-pVDZ level.

ωB97XD/aug-cc-pVDZ	Dipole Moment (D)	HOMO/LUMO Gap (eV)	HOMO/LUMO Gap $\left(\frac{kcal}{mol}\right)$	η (eV)	μ (eV)	σ (eV^−1^)	χ (eV)	ω (eV)
Gas phase	7.83	2.91	67.1	1.45	−5.75	0.69	5.75	11.4
MeOH	12.1	4.45	102.6	2.22	−5.30	0.45	5.30	6.30
DMSO	10.2	3.08	71.0	1.54	−6.00	0.65	6.00	11.7

**Table 4:** HAT/SET energy evaluation. All calculations were performed at the ωB97XD/aug-cc-pVDZ level of theory in the gas phase, MeOH, and DMSO.

	HAT Mechanism		SET Mechanism	
Energies	BDE_OH_ (eV)	${\mathrm{BDE}}_{\mathrm{OH}}\;\left(\frac{kcal}{mol}\right)$	IP (eV)	$\mathrm{IP}\;\left(\frac{kcal}{mol}\right)$
Gas phase	4.16	95.9	7.34	169
MeOH	4.49	104	6.20	143
DMSO	4.00	92.2	5.25	121

## Data Availability

The original contributions presented in this study are included in the article and in the Supplementary Materials. Further inquiries can be directed to the corresponding author.

## Supplementary Materials

The following supporting information can be downloaded at https://www.mdpi.com/article/10.3390/biom15070933/s1, Figures S1–S5: ^1^H, ^13^C NMR, HPLC, ESI-MS, and IR characterization data; Figures S6 and S7, and Table S1: Biological characterization data (antioxidant and antimicrobial assays); Figure S8: UV–vis spectrum predicted by Time-Dependent Self-Consistent Field (TD-SCF) and corresponding data.
