# Supplementary material for: The Synthesis, Characterization, and Biological Evaluation of a Fluorenyl-Methoxycarbonyl-Containing Thioxo-Triazole-Bearing Dipeptide: Antioxidant, Antimicrobial, and BSA/DNA Binding Studies for Potential Therapeutic Applications in ROS Scavenging and Drug Transport
Source: Biomolecules. 2025 Jun 26;15(7):933. doi: 10.3390/biom15070933 (PMC12292336; doi:10.3390/biom15070933)
Supplement: Supplementary file 1 [file biomolecules-15-00933-s001.zip › biomolecules-3648004-supplementary.pdf]

Supporting Information
The Synthesis, Characterization, and Biological Evaluation of a Fluorenyl-Methoxycarbonyl-Containing Thioxo –Triazole –Bearing Dipeptide: Antioxidant, Antimicrobial, and BSA/DNA Binding Studies for Potential Therapeutic Applications in ROS Scavenging and Drug Transport

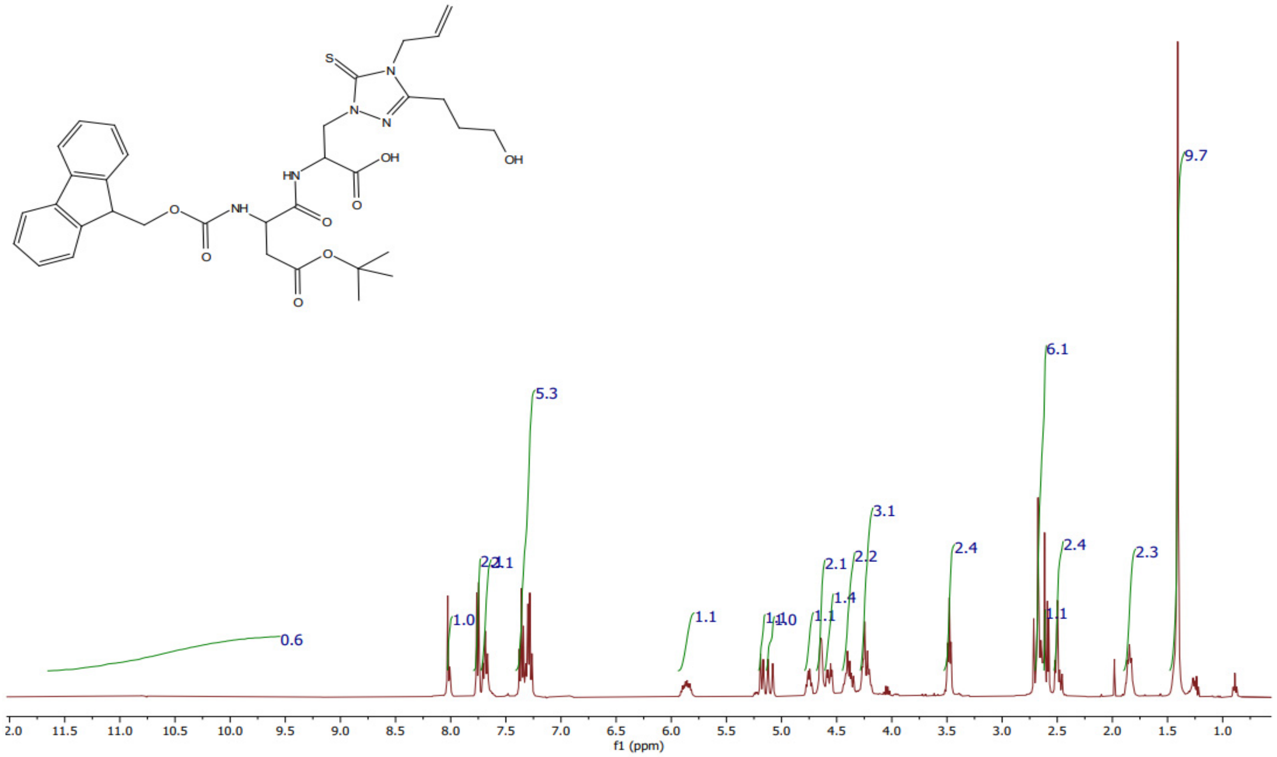

Figure S1. ^{1}H NMR spectrum of (S)-2-((S)-2-((((9H-fluoren-9-yl)methoxy)carbonyl)amino)-4-(tert-butoxy)-4-oxobutanamido)-3-(4-allyl-3-(3-hydroxypropyl)-5-thioxo-4,5-dihydro-1H-1,2,4-triazol-1-yl)propanoic acid (**5**) in DMSO/CCl_4 (1/3).

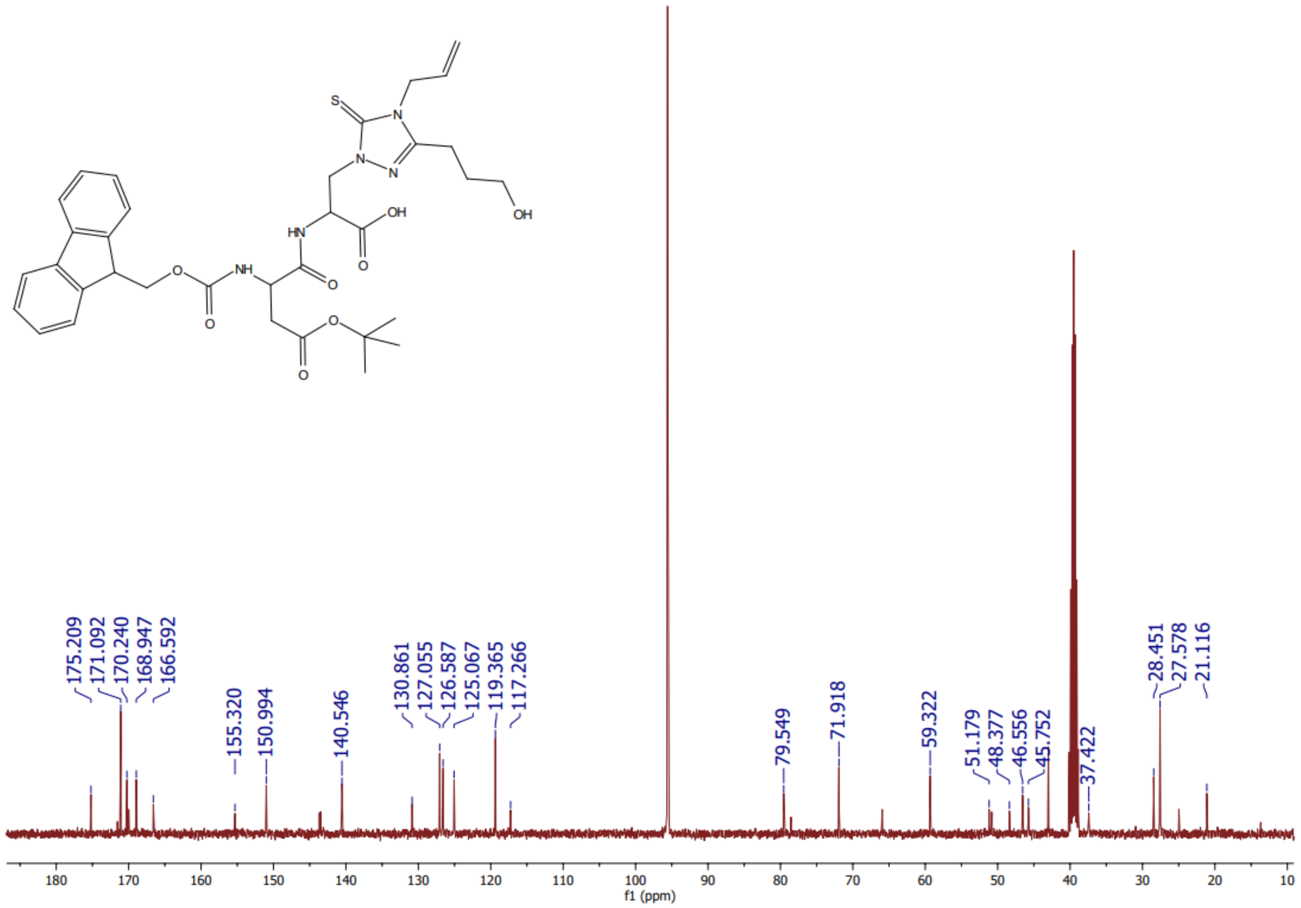

Figure S2. ^{13}C NMR of **5** in DMSO/CCl_4 (1/3).

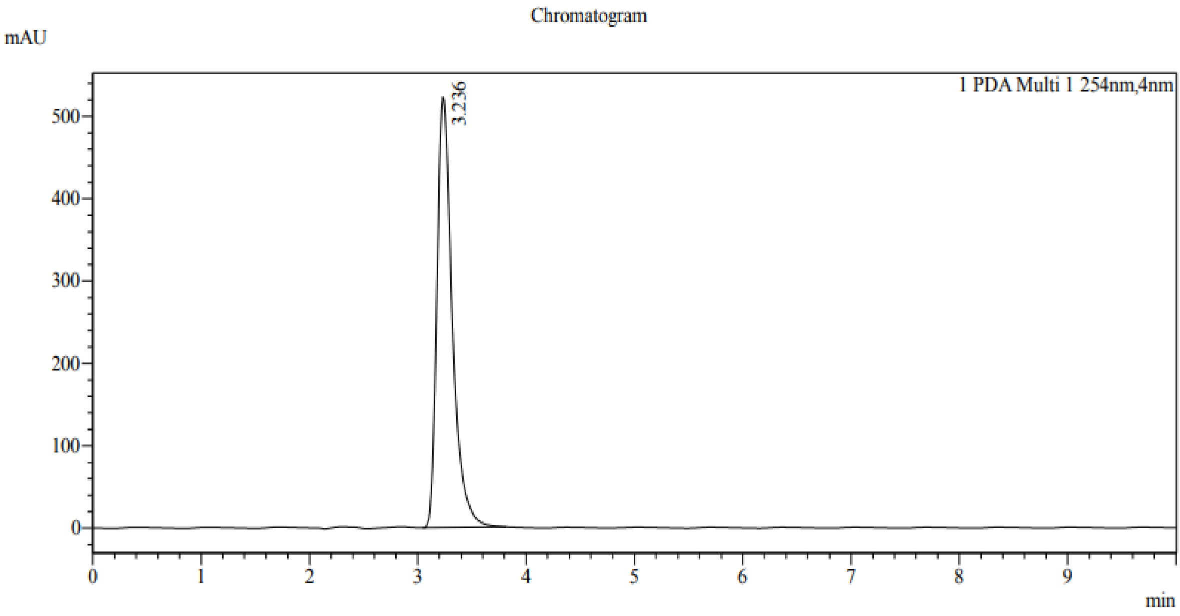

Peak Table

PDA Ch1 254nm

Peak#	Ret. Time	Area	Height
1	3.236	5098964	522766
Total		5098964	522766

Figure S3. HPLC profile for **5**.

MS Chromatogram

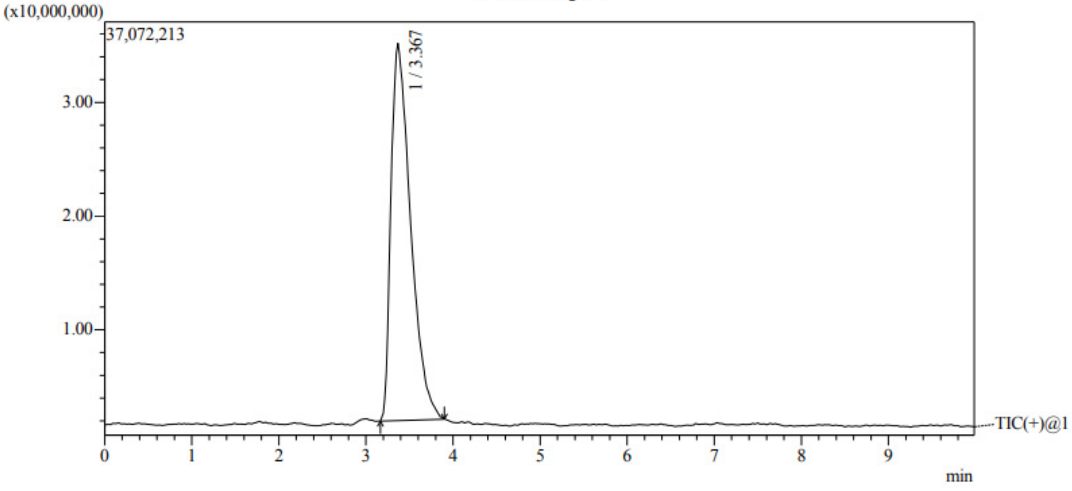

MASS Peak Table TIC

Peak#	Ret. Time	Area	Base Peak m/z
1	3.367	520013873	680.49
Total		520013873	

MS Spectrum

Peak#:1 R.Time:3.367(Scan#:203)
MassPeaks:295
Spectrum Mode:Averaged 3.350-3.383(202-204)
BG Mode:Calc Segment 1 - Event 1

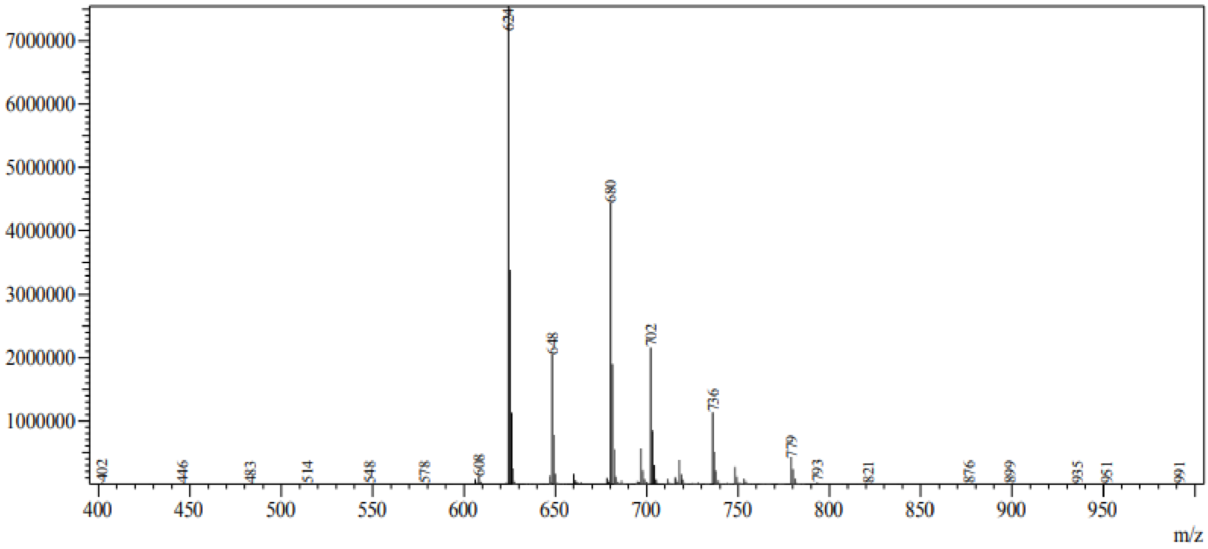

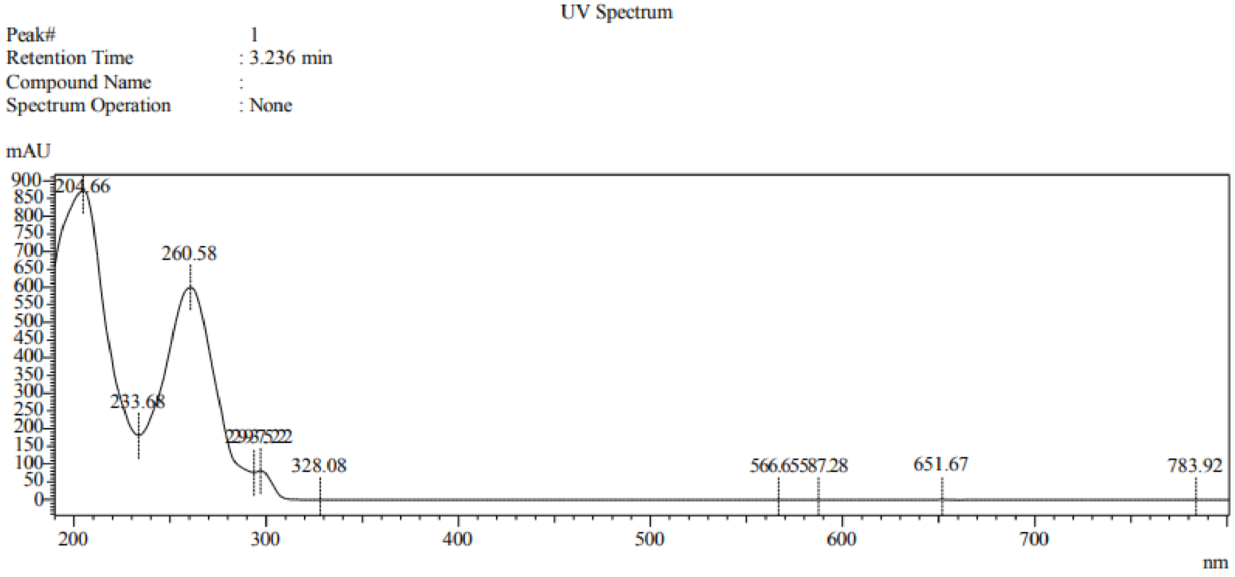

Figure S4. ESI MS and UV analysis of **5**.

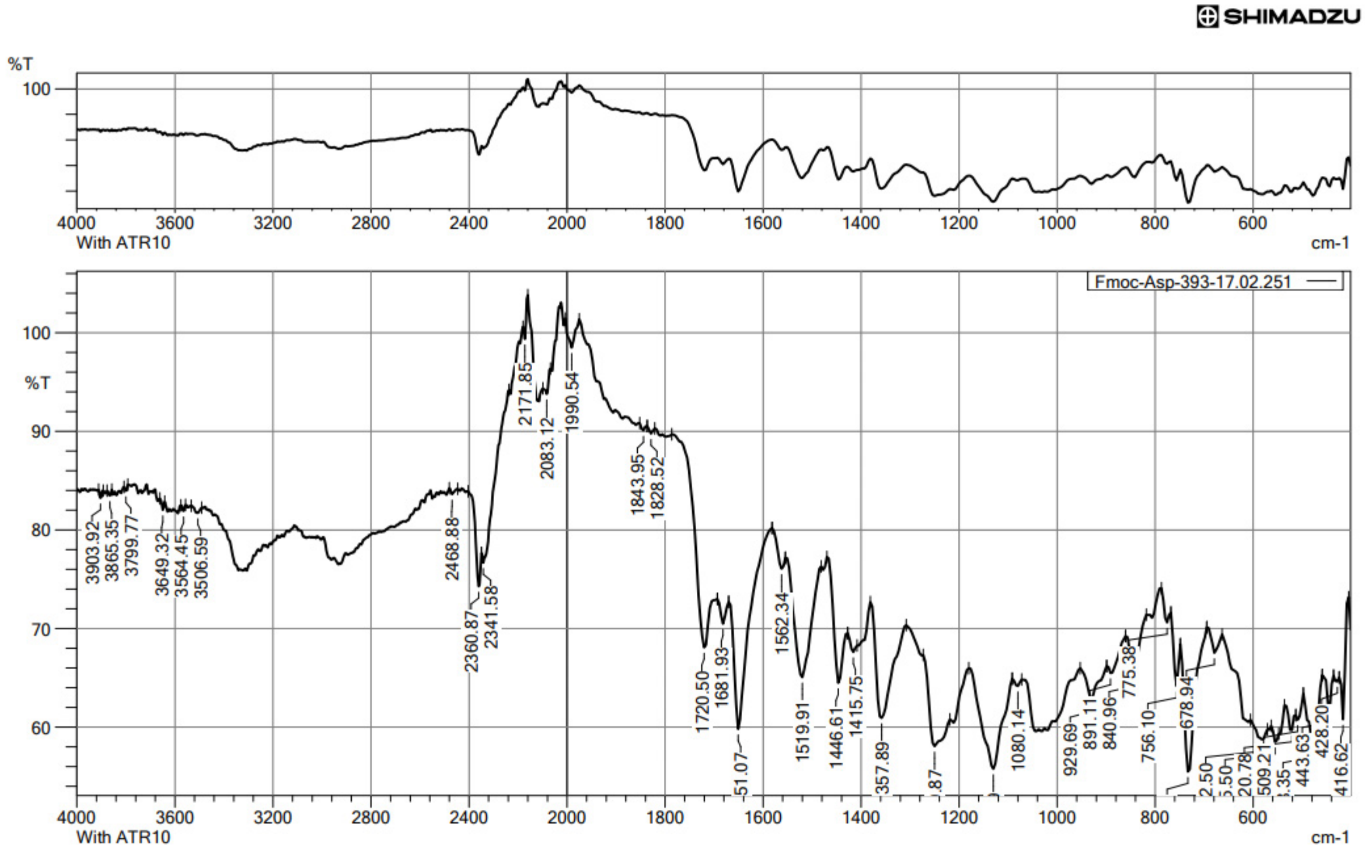

Figure S5. ATR-IR spectrum of **5**, ν, cm−1.

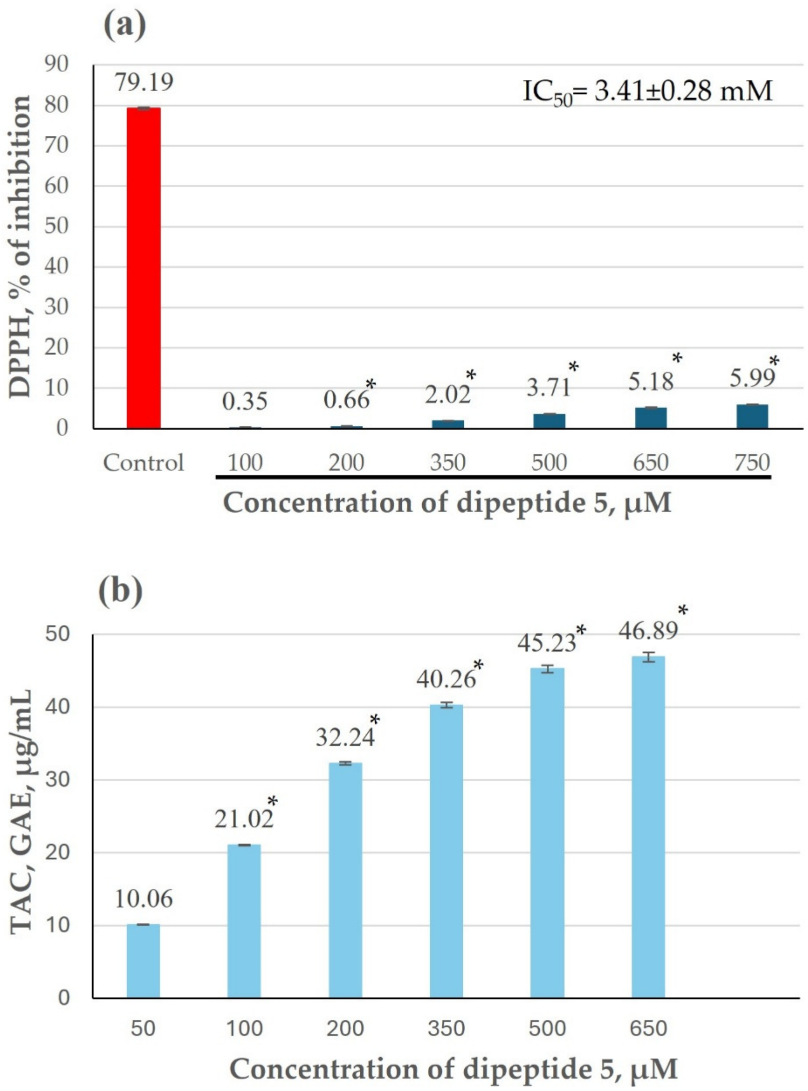

Figure S6. Antioxidant activity of dipeptide **5** solution in methanol, evaluated using DPPH (a) and TAC methods (b). Gallic acid solution (1 μM) in methanol was used as a control (red column). The values in each column represent the mean ± standard deviation of the mean of three independent experiments. Asterisks represent significant differences (*t*-test, * $p < 0.05$) when compared to the antioxidant activity with the lowest concentration of dipeptide **5** in the corresponding experiment.

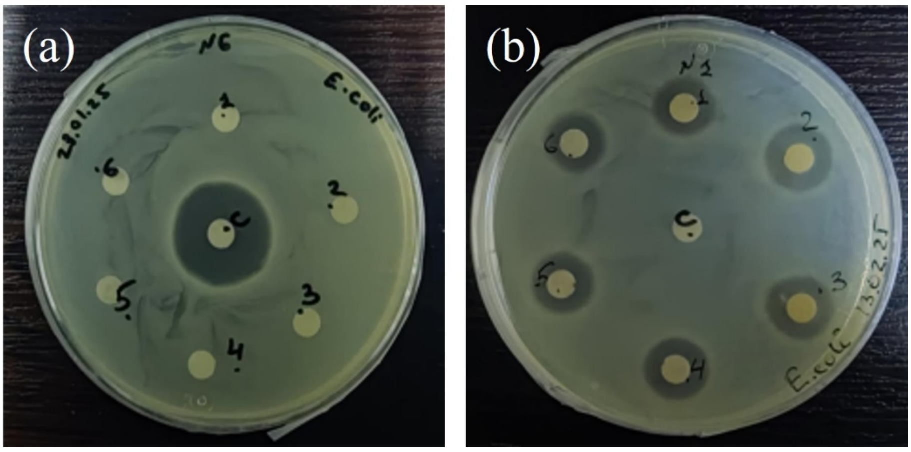

Figure S7. Zones of inhibition of different concentrations of dipeptide **5** and *Paeonia* leaf and root extracts against *E. coli;* **a)**. 1 - dipeptide **5** 5 μg/disk; 2 - dipeptide **5** 10 μg/disk; 3 - dipeptide **5** 15

μg/disk; 4 - dipeptide **5** 25 μg/disk; 5 - - dipeptide **5** 40 μg/disk; 6 – dipeptide **5** 50 μg/disk, C – positive control, 30 μg/disk Amoxicillin; **b)**. 1 - *Paeonia* leaf extract 200 μg/disk; 2 - *Paeonia* leaf extract 200 μg/disk + dipeptide **5** 12 μg/disk; 3 - *Paeonia* leaf extract 200 μg/disk + dipeptide **5** 25 μg/disk; 4 - *Paeonia* root extract 200 μg/disk; 5 - *Paeonia* root extract 200 μg/disk + dipeptide **5** 12 μg/disk; 6 - *Paeonia* root extract 200 μg/disk + dipeptide **5** 25 μg/disk. C – negative control, 10 μl/disk 80% DMSO.

Table S1. Antibacterial activity of different concentrations of dipeptide **5** and *Paeonia* leaf and root extracts against *E. coli.* Asterisks represent significant differences (*t*-test, * $p < 0.05$) when compared to the antibacterial activity of *Paeonia* leaf or root extracts without dipeptide **5**. nd – not detected.

**Concentration of dipeptide 5**	***Paeonia daurica* extract (organ/concentration)**	**Inhibition zone (IZ), mm (mean ± SD)**
5-50 μg/disk	nd	nd
0	Leaf/200 μg/disk	13.6 ± 0.6
12 μg/disk	Leaf/200 μg/disk	14.9 ± 0.3*
25 μg/disk	Leaf/200 μg/disk	15.5 ± 0.4*
0	Root/200 μg/disk	13.9 ± 0.9
12 μg/disk	Root/200 μg/disk	12.4 ±1.0*
25 μg/disk	Root/200 μg/disk	12.0 ± 1.2*

Table S2. Circular Dichroism Data.

**Wavelength (nm)**	**CD BSA + dipeptide 5**	**CD BSA**	**ΔCD (CD Complex - CD BSA)**	**% CD Change (%)**
209	-7.41344	-8.52532	1.11188	13.04
222	-6.73138	-7.70481	0.97343	12.63

[1] Circular Dichroism changes were analyzed for two minima—at 209 nm and 222 nm—of the CD curves for BSA and BSA in complex with dipeptide **5**. ΔCD represents the difference between the CD intensities of the complex and BSA, while %CD Change quantifies the relative change as a percentage.

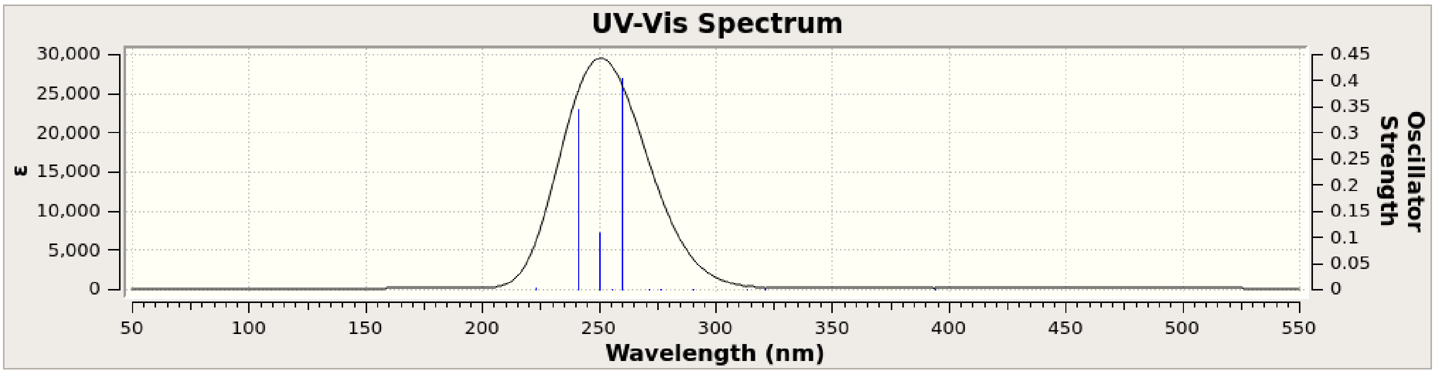

Fig. S8 Time-Dependent Self-Consistent Field (TD-SCF) calculation-predicted UV-vis spectrum; TD-SCF ωB97XD/aug-cc-pVDZ (SCRF=CPCM, MeOH).

*Generic vectorial equation for V_{ESP} in each point of molecule surface:

$$V_{ESP}(\vec{r}) = \sum_N \frac{Z_N}{|\overrightarrow{R_N} - \vec{r}|} - \int \frac{\rho(\overrightarrow{r'})}{|\overrightarrow{r'} - \vec{r}|} d\overrightarrow{r'}$$

Optimized geometry singlet state gas phase

Center Number	Atomic Number	Forces (Hartrees/Bohr) X	Y	Z
1	6	-0.000001403	-0.000000549	0.000000409
2	6	0.000001222	0.000001071	0.000000755
3	6	-0.000000777	0.000001073	-0.000000283
4	8	0.000000244	-0.000000081	-0.000000369
5	8	0.000000502	-0.000000419	0.000000201
6	7	0.000001173	-0.000001311	0.000000900
7	7	-0.000000430	0.000001462	-0.000002015
8	6	0.000000767	-0.000000832	-0.000000238
9	7	0.000000381	-0.000001186	0.000000136
10	6	-0.000001786	-0.000000418	-0.000000615
11	16	0.000000104	-0.000000004	0.000000312
12	6	0.000000745	0.000001827	0.000000722
13	6	-0.000001165	-0.000001829	-0.000001202
14	6	-0.000000621	-0.000000573	-0.000000470
15	8	0.000000316	0.000000326	0.000000479
16	6	-0.000000246	0.000000594	0.000000573
17	6	-0.000000249	0.000001018	-0.000000963
18	6	-0.000000049	-0.000000517	0.000000392
19	6	0.000001016	-0.000002492	0.000001113
20	6	-0.000000731	0.000004971	-0.000002248
21	6	-0.000000027	-0.000002244	0.000000692
22	6	-0.000002267	0.000002670	-0.000001220
23	8	0.000000052	0.000000191	-0.000000835
24	7	0.000000522	-0.000000551	-0.000001203
25	8	-0.000000061	0.000000504	0.000002916
26	8	-0.000000066	0.000000017	0.000001239
27	6	0.000000591	-0.000000836	0.000000959
28	6	0.000000134	-0.000000097	-0.000000685
29	6	-0.000000797	0.000000008	-0.000000808
30	6	0.000000073	-0.000000181	0.000000290
31	7	-0.000000847	-0.000001803	0.000000503
32	6	0.000002160	-0.000000660	-0.000001888
33	8	-0.000001855	0.000000322	0.000000017
34	8	0.000000611	-0.000001202	0.000001023
35	6	-0.000000424	0.000001388	0.000001727
36	6	-0.000000625	-0.000001534	-0.000001203
37	6	-0.000000214	0.000000320	0.000000939
38	6	0.000000702	-0.000000640	0.000000075
39	6	0.000000188	0.000000001	-0.000000421
40	6	-0.000000318	-0.000000078	0.000000404
41	6	0.000000197	-0.000000471	0.000000490

42	6	-0.000000027	0.000000415	-0.000000269
43	6	0.000000299	-0.000000138	-0.000000288
44	6	0.000000039	-0.000000194	0.000000588
45	6	-0.000000405	-0.000000034	0.000000165
46	6	-0.000000365	-0.000000059	0.000000167
47	6	0.000000418	-0.000000027	0.000000414
48	6	0.000000563	0.000000563	-0.000000081
49	1	-0.000000443	-0.000000766	-0.000000405
50	1	-0.000000035	0.000000202	-0.000000003
51	1	-0.000000156	0.000000070	0.000000002
52	1	0.000000154	0.000000502	-0.000000532
53	1	0.000000244	-0.000000162	-0.000000605
54	1	0.000000122	-0.000000246	-0.000000115
55	1	0.000000333	0.000000874	0.000000168
56	1	0.000000006	0.000000051	0.000000093
57	1	0.000000209	0.000000356	-0.000000086
58	1	0.000000350	-0.000000217	-0.000000088
59	1	0.000000170	0.000000371	-0.000000644
60	1	-0.000000358	0.000000214	-0.000000378
61	1	0.000000572	0.000000177	-0.000000378
62	1	0.000000648	0.000000156	-0.000000458
63	1	0.000000186	0.000000182	-0.000000326
64	1	-0.000000126	-0.000000119	-0.000000075
65	1	-0.000000126	-0.000000673	0.000000381
66	1	0.000000093	0.000000328	-0.000000048
67	1	-0.000000017	0.000000198	0.000000007
68	1	-0.000000029	0.000000064	-0.000000073
69	1	-0.000000168	-0.000000128	0.000000471
70	1	0.000000203	-0.000000098	0.000000248
71	1	-0.000000046	-0.000000066	-0.000000045
72	1	0.000000169	-0.000000078	0.000000153
73	1	0.000000459	0.000000177	0.000000070
74	1	0.000000217	-0.000000274	-0.000000288
75	1	-0.000000103	0.000000141	-0.000000119
76	1	0.000000042	0.000000117	-0.000000106
77	1	-0.000000128	-0.000000114	-0.000000097
78	1	0.000000115	0.000000491	0.000001269
79	1	0.000000296	-0.000000190	-0.000000502
80	1	-0.000000336	-0.000000234	0.000000002
81	1	-0.000000256	0.000000903	0.000000217
82	1	0.000000011	0.000000409	0.000000171
83	1	0.000000089	-0.000000150	-0.000000029
84	1	0.000000142	-0.000000086	0.000000082
85	1	0.000000110	0.000000038	0.000000168

86	1	0.000000211	0.000000012	0.000000237
87	1	0.000000055	-0.000000093	0.000000243
88	1	0.000000119	-0.000000047	0.000000085
89	1	-0.000000266	-0.000000078	0.000000042

Optimized geometry singlet state CPCM, solvent MeOH

Center Number	Atomic Number	Forces (Hartrees/Bohr) X	Y	Z
1	6	0.000002896	-0.000000508	0.000001033
2	6	-0.000000363	0.000001489	0.000000151
3	6	0.000000001	0.000000070	-0.000001459
4	8	-0.000001828	0.000000150	-0.000001328
5	8	-0.000001713	0.000000804	-0.000000911
6	7	0.000000083	-0.000000275	-0.000003690
7	7	0.000000340	0.000000028	0.000001104
8	6	0.000000217	0.000001024	-0.000001885
9	7	-0.000000643	0.000000129	-0.000001702
10	6	0.000001358	0.000001760	0.000003503
11	16	-0.000000329	0.000000124	-0.000001581
12	6	-0.000000115	0.000000561	-0.000001593
13	6	0.000000368	0.000000187	-0.000000917
14	6	0.000000407	0.000000975	-0.000001413
15	8	0.000000141	0.000000536	-0.000001707
16	6	0.000001241	0.000001062	-0.000002220
17	6	0.000000257	0.000000334	-0.000001126
18	6	-0.000000705	0.000001003	-0.000002184
19	6	0.000000828	-0.000001618	-0.000002036
20	6	-0.000001984	0.000000941	0.000001593
21	6	-0.000001608	-0.000000495	-0.000000386
22	6	-0.000000751	-0.000000154	0.000000456
23	8	-0.000001557	0.000001316	0.000001015
24	7	-0.000001093	-0.000000074	-0.000000124
25	8	0.000001384	-0.000000471	-0.000000646
26	8	0.000001486	0.000000576	0.000000166
27	6	0.000000106	-0.000001231	0.000001406
28	6	-0.000000029	-0.000000895	0.000000817
29	6	0.000000714	-0.000000924	0.000000768
30	6	0.000000172	-0.000000091	0.000000885
31	7	-0.000001867	0.000000871	-0.000000335
32	6	-0.000000131	0.000001837	0.000001504

33	8	-0.000000904	-0.000001278	0.000000622
34	8	-0.000002100	-0.000002016	0.000002604
35	6	0.000000101	-0.000000771	-0.000000075
36	6	0.000000370	-0.000002274	-0.000001625
37	6	0.000000760	0.000001338	0.000001219
38	6	-0.000000087	-0.000000235	0.000001615
39	6	0.000000166	-0.000000257	-0.000000050
40	6	0.000000623	-0.000000390	0.000000349
41	6	0.000000816	-0.000000346	0.000000829
42	6	0.000000578	-0.000001341	0.000000044
43	6	-0.000000557	-0.000000252	0.000001206
44	6	0.000000624	-0.000000617	0.000000855
45	6	0.000000258	-0.000000535	0.000001203
46	6	-0.000000402	-0.000000946	0.000001549
47	6	-0.000000199	-0.000000562	0.000001283
48	6	-0.000000421	-0.000000363	0.000001901
49	1	-0.000000676	0.000000282	-0.000000531
50	1	0.000000252	-0.000000026	-0.000000455
51	1	0.000000151	0.000000144	-0.000000437
52	1	-0.000000572	0.000000610	-0.000000591
53	1	0.000000491	0.000000628	-0.000001687
54	1	0.000000896	0.000000462	-0.000000835
55	1	0.000000110	0.000000763	-0.000001397
56	1	0.000000246	0.000000511	-0.000001022
57	1	0.000000428	0.000000558	-0.000001484
58	1	0.000000434	0.000000746	-0.000001625
59	1	0.000000224	0.000000967	-0.000001727
60	1	-0.000000359	0.000001069	-0.000001732
61	1	-0.000000103	0.000000509	-0.000001439
62	1	0.000000007	0.000001327	-0.000001452
63	1	0.000000634	0.000001012	-0.000002061
64	1	-0.000000138	0.000001004	-0.000001970
65	1	-0.000000643	0.000000542	0.000000631
66	1	0.000000047	0.000000730	0.000001354
67	1	0.000000024	-0.000000118	0.000000275
68	1	-0.000000525	0.000001014	0.000000529
69	1	0.000000012	-0.000000746	0.000001256
70	1	0.000000437	-0.000000733	0.000001249
71	1	-0.000000000	-0.000000651	0.000001205
72	1	-0.000000314	-0.000000709	0.000000723
73	1	0.000000393	-0.000000704	0.000000859
74	1	-0.000000185	-0.000000482	0.000001114
75	1	0.000000174	-0.000000330	0.000000640
76	1	0.000000110	-0.000000429	0.000000570

77	1	0.000000785	-0.000000649	0.000000918
78	1	0.000000359	-0.000001108	0.000000298
79	1	-0.000000985	-0.000000268	0.000000275
80	1	-0.000000657	0.000000178	0.000001361
81	1	-0.000000340	-0.000000383	0.000001009
82	1	0.000000254	0.000000168	-0.000000854
83	1	0.000000530	-0.000000110	-0.000000124
84	1	0.000000847	-0.000000367	0.000000091
85	1	0.000000624	-0.000000487	0.000000541
86	1	0.000000507	-0.000000788	0.000001118
87	1	0.000000258	-0.000001003	0.000001582
88	1	-0.000000170	-0.000000843	0.000001666
89	1	-0.000000476	-0.000000488	0.000001470

Optimized geometry singlet state CPCM, solvent DMSO

Center Number	Atomic Number	Forces (Hartrees/Bohr) X	Y	Z
1	6	-0.000000319	-0.000001047	0.000001600
2	6	0.000000293	-0.000002145	-0.000000294
3	6	0.000000865	-0.000000487	0.000000722
4	8	0.000000667	0.000001621	-0.000001135
5	8	0.000000365	-0.000000233	-0.000000160
6	7	-0.000000674	0.000001145	-0.000001218
7	7	-0.000000873	-0.000001833	-0.000000553
8	6	0.000002390	0.000000741	-0.000000640
9	7	-0.000001493	-0.000000230	0.000003504
10	6	-0.000001348	-0.000000592	-0.000000859
11	16	-0.000000112	0.000000284	0.000000172
12	6	-0.000001499	-0.000001933	0.000000166
13	6	0.000000479	0.000000654	0.000000665
14	6	-0.000000790	0.000000504	0.000000645
15	8	0.000000819	-0.000000258	-0.000000565
16	6	0.000001277	-0.000000498	-0.000001234
17	6	-0.000000717	-0.000000468	0.000000290
18	6	-0.000000532	-0.000001352	0.000000674
19	6	0.000001733	0.000002166	-0.000002053
20	6	-0.000003219	-0.000001595	0.000001882
21	6	0.000002356	-0.000001098	0.000000466
22	6	-0.000001523	-0.000000476	0.000001221
23	8	-0.000001242	-0.000001701	0.000001900

24	7	-0.000000809	-0.000001061	0.000000947
25	8	0.000000857	0.000001462	-0.000000937
26	8	0.000000956	0.000000764	0.000000301
27	6	-0.000000046	-0.000000106	-0.000000426
28	6	0.000000314	-0.000000191	-0.000000028
29	6	0.000000339	-0.000000088	-0.000001087
30	6	0.000000672	0.000000312	0.000000775
31	7	0.000000978	0.000001337	-0.000001026
32	6	-0.000000636	0.000000132	-0.000001388
33	8	-0.000000226	0.000000566	0.000000472
34	8	0.000001386	-0.000002637	0.000001628
35	6	-0.000001089	0.000002475	-0.000000764
36	6	-0.000000348	0.000000338	0.000000130
37	6	0.000000308	0.000000674	0.000000062
38	6	-0.000000406	0.000000216	-0.000000134
39	6	0.000000039	0.000000185	0.000000060
40	6	-0.000000568	0.000000051	-0.000000003
41	6	0.000000294	0.000000075	0.000000225
42	6	-0.000000345	0.000000146	0.000000056
43	6	-0.000000607	0.000000083	-0.000000278
44	6	0.000000035	0.000000862	0.000000186
45	6	-0.000000252	0.000000219	0.000000062
46	6	0.000000422	0.000000847	-0.000000159
47	6	-0.000001073	0.000000021	0.000000091
48	6	0.000000708	-0.000000008	-0.000000007
49	1	0.000000642	0.000001076	0.000000449
50	1	-0.000000346	0.000000516	0.000000431
51	1	0.000000298	-0.000000246	-0.000000879
52	1	-0.000000287	0.000000166	0.000000047
53	1	0.000000368	-0.000000526	-0.000000504
54	1	0.000000169	0.000000214	-0.000000757
55	1	-0.000000277	0.000000116	-0.000000550
56	1	0.000000339	-0.000000861	-0.000000285
57	1	-0.000000165	-0.000000078	-0.000000028
58	1	-0.000000159	-0.000000410	-0.000000476
59	1	-0.000001086	-0.000000376	0.000000821
60	1	-0.000000504	-0.000000185	0.000000056
61	1	0.000000360	-0.000000339	-0.000000239
62	1	0.000000617	0.000000323	0.000000123
63	1	-0.000000027	-0.000000340	-0.000000211
64	1	-0.000000218	-0.000000336	0.000000197
65	1	-0.000000387	0.000000265	-0.000000710
66	1	0.000000138	0.000000202	-0.000000647
67	1	-0.000000372	0.000000807	-0.000000422

68	1	0.000000322	0.000001054	-0.000000321
69	1	0.000000402	-0.000000008	-0.000000069
70	1	0.000000346	0.000000234	-0.000000107
71	1	0.000000324	0.000000078	0.000000089
72	1	0.000000034	-0.000000234	0.000000021
73	1	0.000000323	-0.000000254	0.000000342
74	1	0.000000343	0.000000117	0.000000053
75	1	0.000000211	0.000000130	-0.000000123
76	1	0.000000044	-0.000000183	-0.000000147
77	1	0.000000450	0.000000092	-0.000000208
78	1	0.000000462	-0.000000250	0.000000253
79	1	-0.000000014	-0.000000465	0.000000607
80	1	0.000000356	-0.000000450	-0.000000400
81	1	0.000000100	0.000000276	-0.000000111
82	1	0.000000055	0.000000105	-0.000000097
83	1	-0.000000082	0.000000092	-0.000000031
84	1	-0.000000175	0.000000155	-0.000000032
85	1	-0.000000247	0.000000423	-0.000000035
86	1	-0.000000023	0.000000150	-0.000000126
87	1	-0.000000166	0.000000423	0.000000002
88	1	-0.000000109	0.000000407	0.000000060
89	1	0.000000135	0.000000277	0.000000009
